# Chemo-enzymatic Approach to (*R*)‑Perillaldehyde: Improving the Sustainability of the Reaction Steps with the Principles of Green Chemistry

**DOI:** 10.1021/acs.oprd.5c00340

**Published:** 2025-11-24

**Authors:** Federico Acciaretti, Celeste Nobbio, Natale Crisafulli, Martina Arosio, Francesco G. Gatti, Fabio Parmeggiani, Elisabetta Brenna

**Affiliations:** Dipartimento di Chimica, Materiali ed Ingegneria Chimica “Giulio Natta”, 18981 Politecnico di Milano, Piazza Leonardo da Vinci 32, 20133 Milano, Italy

**Keywords:** sustainability, epoxide rearrangement, oxidation, alcohol dehydrogenase, chemoselectivity

## Abstract

In this work, a new chemo-enzymatic synthesis of (*R*)-perillaldehyde ((*R*)-**1**, 98% ee) was developed by progressively improving the sustainability of the reaction steps. The key transformation is the oxidation of (*R*)-perillyl alcohol ((*R*)-**2**), catalyzed by a recombinant alcohol dehydrogenase from *Geobacillus stearothermophilus* (ADH-hT), used as cell-free extract (CFE), in the presence of acetone as a sacrificial substrate. Alcohol (*R*)-**2** is obtained in a mixture (44% by NMR analysis) with secondary alcohols **4** and **5** in a two-step sequence starting from the rearrangement of (4*R*)-limonene oxides catalyzed by aluminum isopropylate in toluene and subsequent allylic rearrangement of the intermediates by S_N_2′ displacement in aqueous acetone. Perillyl alcohol is recovered by column chromatography and oxidized with ADH-hT as a catalyst to afford (*R*)-perillaldehyde (98% ee), which is isolated in pure form by distillation under reduced pressure (22% isolated yield from limonene oxides). When the reaction is performed on the crude mixture containing perillyl alcohol together with the secondary alcohols **4** and **5** as side products, complete chemoselectivity toward the oxidation of the primary alcohol is observed. Thus, we also describe the chemoselective oxidation of alcohol **2** in this mixture (44% by NMR analysis) by means of ADH-hT and subsequent isolation of the corresponding aldehyde by formation of the Bertagnini adduct. A comparison between these two routes and those described in the literature is herein discussed.

## Graphical abstract

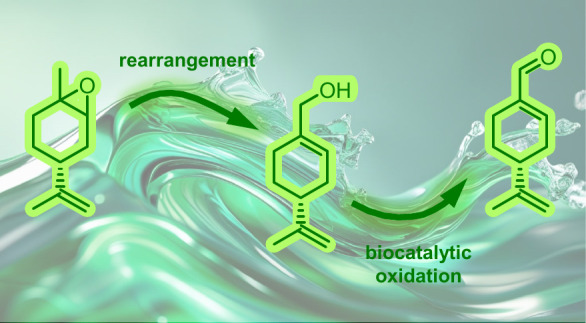

## 1 Introduction

For an ongoing research work devoted to the synthesis of fragrances, we planned to employ as a starting material the *R* enantiomer of perillaldehyde ((*R*)-**1**, Figure 1), which cannot be found on the market. Besides, the corresponding (*R*)-perillyl alcohol ((*R*)-**2**), once available, is no longer commercialized. Aldehyde (*R*)-**1** is much less abundant in nature than the corresponding *S* enantiomer, which is the main constituent of the essential oil of *Perilla frutescens*.^1^ Only recently, the *R* enantiomer was identified in the essential oil obtained starting from the dried fruits of *Ammodaucus leucotrichus* L. (hairy cumin) and found to be a ferroptosis inducer with relevant clinical potential for acute myeloid leukemia.^2^ (*R*)-**1** is also a key intermediate in the synthesis of some cannabinoid derivatives^3,4^ and biologically active natural compounds.^5,6^

**Figure 1:**
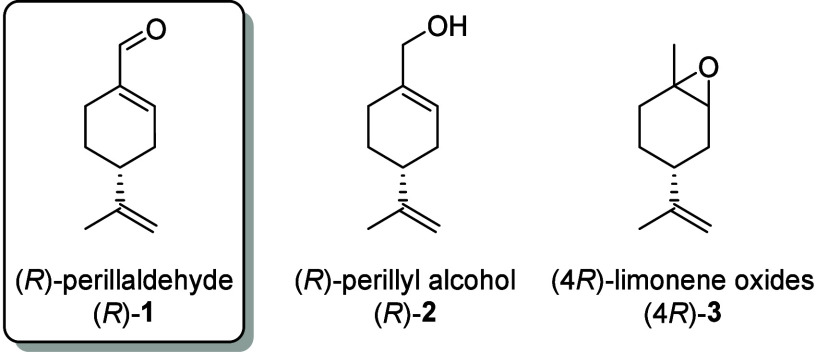
(*R*)-Perillaldehyde and related precursors.

A literature search highlighted that (4*R*)-limonene oxides ((4*R*)-**3**) are the most attractive starting material for the preparation of (*R*)-**1** and (*R*)-**2**. Tius and Kerr reported the procedure depicted in Scheme 1 to synthesize (*R*)-**1** starting from (4*R*)-**3**.^7^ Rearrangement of the oxirane ring with an excess of methylmagnesium cyclohexylisopropylamide (4 equiv) afforded the allylic alcohols **4**, which were then converted by reaction with phenylsulfenyl chloride into a rearranged sulfoxide derivative. The latter was submitted first to Pummerer rearrangement and then to treatment with aqueous mercuric chloride to give a final mixture containing (*R*)-perillaldehyde with a 21% yield of a sulfide impurity.

**Scheme 1:**
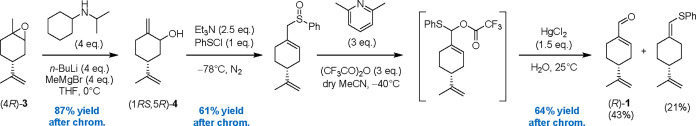
Known Procedure for the Preparation of (*R*)-Perillaldehyde^7^

Wong et al. prepared the two enantiomers of perillaldehyde by rearrangement of β-pinene oxide with ammonium nitrate in nitromethane at 80 °C.^8^ Unfortunately, the preparation of (*R*)-**1** requires (+)-β-pinene, which is not commercially available and has to be prepared by a rather complex procedure from (+)-α-pinene. In 2008, Serra et al.^9^ recovered (*R*)-**1** as a byproduct in the synthetic procedure of the cooling agent 1-hydroxy-2,9-cineole.

A few reports describe the preparation of perillaldehyde by chemical oxidation of (*R*)-perillyl alcohol,^10–12^ either using the commercial product once available on the market or without disclosing any information on the procedure used to prepare the starting alcohol. Even enzymatic oxidations are limited, and the absolute configuration of the starting alcohol is not clearly reported in the papers.^13–15^

Only a few synthetic approaches to alcohol (*R*)-**2** have been reported in the literature. Allylic hydroxylation of the methyl group of (*R*)-limonene using P450 monooxygenase has been studied, but despite good results, none of these procedures has been carried out on a preparative scale.^16,17^ Complex mixtures containing (*R*)-perillyl alcohol can be obtained by chemical epoxidation of (*R*)-limonene and further rearrangement, using either a 60% w/w solution of hydrogen peroxide in the presence of zeolite-type catalysts^18^ or a combination of *tert*-butyl hydroperoxide and molybdenum catalysts.^19^ An interesting approach to (*R*)-perillyl alcohol was reported in 2014 (Scheme 2, route (a)), exploiting the lithium diisopropylamide (LDA)-promoted rearrangement of (4*R*)-**3** to afford a mixture of the two diastereoisomers of allylic alcohol (1*RS*,5*R*)-**4**.^20^ The acetyl derivatives of these compounds were submitted to isomerization catalyzed by Pd­(PPh_3_)_4_ at 110 °C to give invariably a final mixture containing, after saponification, 65% perillyl alcohol and 35% starting alcohols **4**. A more straightforward approach was reported a few years later by McAulay and Clark^21^ (Scheme 2, route (b)). They employed an S_N_2′ allylic rearrangement of the mesylate derivative of intermediate **4** promoted by treatment of the reaction mixture with a saturated solution of sodium hydrogen carbonate during the workup process following mesylation to obtain (*R*)-**2** as a final compound.

**Scheme 2:**
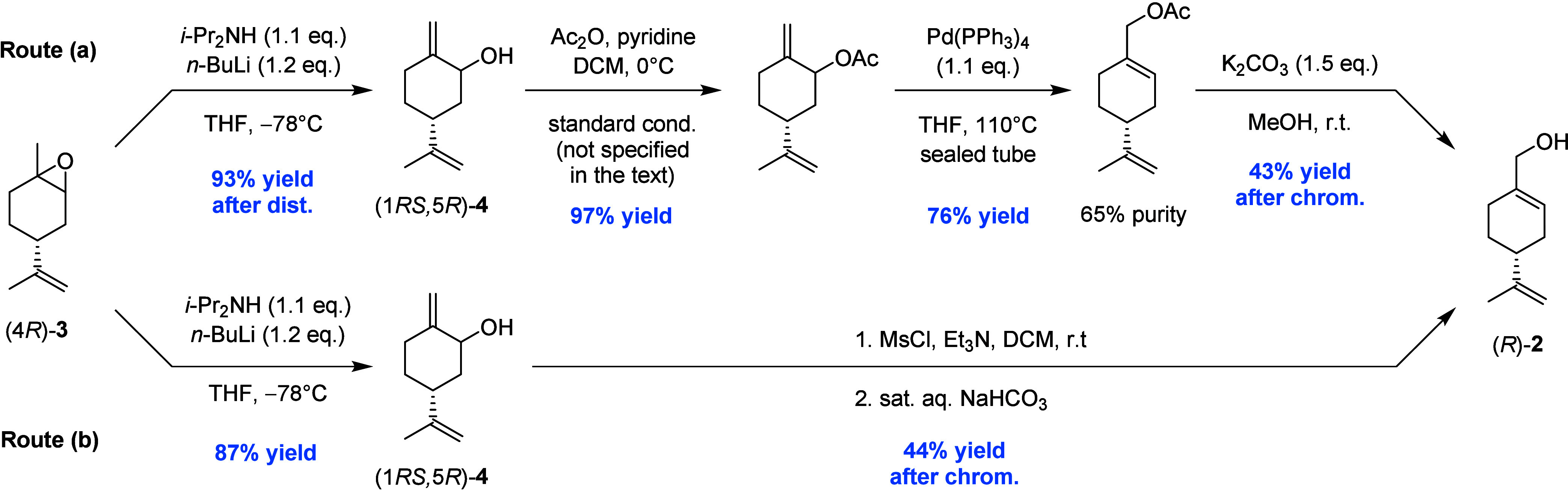
Known Procedures for the Preparation of (*R*)-Perillyl Alcohol^20,21^

Thus, we decided to study a new route to (*R*)-perilladehyde with the aim of (i) avoiding problematic reagents requiring specific reaction conditions (e.g., completely anhydrous conditions under inert atmosphere or heating in sealed reactors), (ii) limiting the use of chlorinated solvents, and (iii) improving the overall sustainability of the process, still using limonene oxides as the starting material. (*R*)-Limonene is largely available, recovered from citrus peels as a side product of the juice industry. The *ca*. 6:4 mixture of the two corresponding monoepoxides is a low-cost commercial product prepared by classical epoxidation of (*R*)-limonene. It is a FEMA-GRAS ingredient employed in the flavor and fragrance industry.^22^ It has recently gained major attention since it has been successfully employed to produce polycarbonates by copolymerization with carbon dioxide and polyesters by copolymerization with anhydrides.^23^ In the last years, there has been an increasing focus on research into the epoxidation of natural terpenes, including limonene, using environmentally benign oxidants like hydrogen peroxide.^24^ Recently, the use of a lipase (*Candida antarctica* lipase fraction B, NS 88011) to generate *in situ* the necessary peracid (in this case, peroctanoic acid) by perhydrolysis of the corresponding acid in the presence of 30% aqueous H_2_O_2_ has been optimized in a fed-batch reactor for (*R*)-limonene epoxidation.^25^

## 2 Experimental Section

### 2.1 General Methods

Chemicals and solvents were purchased from Merck Life Science s.r.l. and Zentek s.r.l. and used without further purification. TLC analyses were performed on Macherey-Nagel precoated TLC sheets (Polygram SIL G/UV_254_) purchased from Chimikart s.r.l. (Naples, Italy). The chromatographic separations were carried out on a PuriFlash XS-420+ system (Interchim) using Purezza-Daily Standard Flash cartridges (Sepachrom, Italy). GC analyses for the determination of the enantiomeric excess were performed by using a MEGA-DEX B-04 column (30 m × 0.25 mm × 0.25 μm), H_2_ as the carrier gas at a flow rate of 0.8 mL min^–1^, an injector temperature of 250 °C, a detector temperature of 250 °C, and the following temperature program: 105 °C (50 min)/90 °C min^–1^/200 °C (5 min). ^1^H and ^13^C NMR spectra were recorded on a 400 MHz spectrometer in CDCl_3_ solution at room temperature, unless otherwise specified. The chemical shift scale was based on internal tetramethylsilane. GC/MS analyses were performed using an HP-5MS column (30 m × 0.25 mm × 0.25 μm, Agilent Technologies Italia S.p.A., Italy). The following temperature program was employed: 60 °C (1 min)/6 °C min^–1^/150 °C (1 min)/12 °C min^–1^/280 °C. Optical rotations [α]_D_ were determined on a digital automatic polarimeter at 589 nm (sodium D line) and are given at 20 °C in deg cm^3^ g^–1^ dm^–1^.

### 2.2 Production of ADH-hT from *Geobacillus stearothermophilus* in *Escherichia coli*

A single colony of *E. coli* BL21­(DE3) carrying the desired plasmid was used to inoculate LB medium (5 mL) supplemented with 50 μg/mL kanamycin, which was grown overnight at 37 °C and 180 rpm. The starter culture was used to inoculate LB medium (500 mL, pH 7.0–7.5 unadjusted) containing 50 μg/mL kanamycin, which was incubated at 37 °C and 220 rpm until an OD value of 0.6–0.8 was reached. Induction was started by adding 0.1 mM IPTG and incubating at 20 °C and 180 rpm for 18 h. Cells were then harvested by centrifugation (5000 rpm, 20 min, 4 °C), washed with KP_i_ buffer (50 mM, pH 7.0) and harvested again by centrifugation (5000 rpm, 20 min, 4 °C). The cell pellet was resuspended with the desired amount of KP_i_ buffer (50 mM, pH 7.0), disrupted by sonication (20 s on, 20 s off, 20 cycles, 4 °C), and centrifuged (17,000 rpm, 20 min, 4 °C). Supernatant was aliquoted and stored at −20 °C. The activity of ADH-hT CFE (0.30 g_CWW_/mL, CWW = cell wet weight) was determined by a spectrophotometric assay, measuring NADH formation at 340 nm upon the oxidation of ethanol.

The 1 mL reaction mixtures contained 945 μL of KP_i_ buffer (50 mM, pH 8.0), 20 μL of NAD^+^ (10 mM), 20 μL of (*R*)-perillyl alcohol (100 mM in DMSO), and 15 μL of ADH-hT CFE. The absorbance at 340 nm was monitored for 5 min at 30 °C. The activity of ADH-hT CFE was 19.3 U/mL.

### 2.3 Reaction of (*R*)-Limonene Oxides with Al­(O-*i*-Pr)_3_

Al­(O-*i*-Pr)_3_ (5.26 mmol, 1.08 g) was slowly added to a warm solution (30–35 °C) of (*R*)-limonene oxides (105.2 mmol, 16.0 g, *trans*/*cis* 56:44) in toluene (100 mL). The reaction mixture was further refluxed for 3 h, cooled, and quenched with a 25% acetic acid solution. The organic phase was recovered, washed with brine, and dried. After removal of the solvent under reduced pressure, a residue (14.7 g, 92%) was obtained showing the following molar composition (^1^H NMR, average of three runs): 8% *trans*-**3**, 44% (1*R*,5*R*)-**4**, 22% (1*S*,5*R*)-**4**, 7% (1*R*,5*R*)-**5**, 16% (1*S*,5*R*)-**5**, and 4% carvone. Here are the NMR signals employed for product identification and quantification: ^**1**^ **H NMR** (400 MHz, CDCl_3_, ppm) δ: C*H*=C: 6.78–6.73 (m, carvone), 5.61–5.57 (m, (1*S*,5*R*)-**5**),^26^ 5.52–5.48 (m, (1*R*,5*R*)-**5**);^27^ C(2)=C*H*_*2*_: 4.95 (q, *J* = 1.7 Hz) and 4.79 (q, *J* = 1.7 Hz) for (1*R*,5*R*)-**4**, 4.86–4.84 (m) and 4.78–4.76 (m) for (1*S*,5*R*)-**4**); C*H*–OH: 4.37 (t, *J* = 3.0 Hz, (1*S*,5*R*)-**4**),^28^ 4.23–4.16 (m, (1*R*,5*R*)-**5**), 4.13–4.06 (m, (1*R*,5*R*)-**4**), 4.04–4.01 (m, (1*S*,5*R*)-**5**); C*H*–O: 2.99 (d, *J* = 2.35 Hz, *trans*-**3**). ^**13**^ **C NMR** (101 MHz, CDCl_3_, ppm) δ: *C*HOH: 72.49 (1*S*,5*R*)-**4**), 72.66 (1*R*,5*R*)-**4**, 71.02 (1*R*,5*R*)-**5**, 68.66 (1*S*,5*R*)-**5**. **GC/MS** (EI): (4*R*)-**3**: *t*_r_ = 7.48 min, *m*/*z* (%) = 152 (M^+^, 15), 134 (22), 108 (48), 91 (100); (1*S*,5*R*)-**4**: *t*_r_ = 8.61 min, *m*/*z* (%) = 152 (M^+^, 0.5), 134 (50), 119 (67), 91 (100); (1*S*,5*R*)-**5**: *t*_r_ = 9.31 min, *m*/*z* (%) = 152 (M^+^, 5), 134 (30), 119 (60), 91 (100); (1*R*,5*R*)-**4**: *t*_r_ = 9.54 min, *m*/*z* (%) = 152 (M^+^, 5), 134 (50), 119 (54), 91 (100); (1*R*,5*R*)-**5**: *t*_r_ = 9.59 min, *m*/*z* (%) = 152 (M^+^, 0.5), 134 (47), 119 (62), 91 (100); carvone: *t*_r_ = 9.83 min, *m*/*z* (%) = 150 (M^+^, 14), 108 (41), 82 (100).

### 2.4 Allylic Rearrangement by S_N_2′ Displacement of the Mesylates of Alcohols **4** and **5**

To a solution of the mixture (11.8 g, 77.6 mmol) was added the mixture recovered from the previous reaction (containing 66% *cis*- and *trans*-**4**) in acetone (70 mL). In the presence of Et_3_N (155.2 mmol, 21.6 mL), mesyl chloride (100.8 mmol, 7.8 mL) was added dropwise at 0 °C. After stirring at room temperature until complete conversion of the alcohols into the corresponding mesylates (4 h), a saturated aqueous solution of NaHCO_3_ (100 mL) was added. The mixture was stirred for 5 h, then extracted with 2-methyl-THF. Distillation of the solvent under reduced pressure left a residue (10.7 g) containing 40% perillyl alcohol (NMR analysis), which was divided in two portions. One portion (3.5 g) was chromatographed on a silica gel column eluting with hexane and increasing amount of EtOAc (from 96:4 hexane/EtOAc to 60:40 hexane/EtOAc) to afford (*R*)-**2** (1.29 g, 33.5% calculated from the corresponding amount of starting mixture) as a pure compound. ^**1**^ **H NMR** (400 MHz, CDCl_3_, ppm) δ: 5.66–5.61 (1H, m, C*H*=), 4.68–4.62 (2H, m, C*H*_*2*_=), 3.99 −3.89 (2H, m, C*H*_*2*_–OH), 2.15–2.00 (4H, m, hydrogens of the cyclohexene ring), 1.96–1.84 (1H, m, hydrogen of the cyclohexene ring), 1.84–1.74 (1H, m, hydrogen of the cyclohexene ring), 1.67 (3H, t, *J* = 1.1 Hz, C*H*_*3*_), 1.48–1.35 (1H, m, hydrogen of the cyclohexene ring). ^**13**^ **C NMR** (101 MHz, CDCl_3_, ppm) δ: 149.94, 137.41, 122.64, 108.81, 67.43, 41.30, 30.56, 27.63, 26.26, 20.93. **GC/MS** (EI): (*R*)-**2**: *t*_r_ = 11.09 min, *m*/*z* (%) = 152 (M^+^, 3), 134 (26), 119 (100), 91 (100). [α]_D_ = +83 (*c* 1.0, CHCl_3_), lit. ref 20 [α]_D_ = +84 (*c* 1.8, CHCl_3_).

Another portion (7.2 g) was submitted to bulb-to-bulb distillation (10 mmHg, 120–125 °C) to give the following mixture (6.42 g): 44% (*R*)-**2**, 3% carvone, 25% *trans*-**5**, 2% *cis*-**5**, 4% *cis*-**4**, and 22% *trans*-**4** by ^1^H NMR. The following signals of (*R*)-**2** can be detected in the NMR spectra: ^**1**^ **H NMR** (400 MHz, CDCl_3_, ppm) δ: 5.66–5.61 (m, C*H*=C), 3.99 −3.89 (m, C*H_2_* OH); ^**13**^ **C NMR** (101 MHz, CDCl_3_) δ: 149.81, 137.30, 122.29, 108.68, 67.06, 41.22, 30.46, 27.54, 26.15, 20.81.

### 2.5 ADH-Mediated Oxidation of the Mixture of 44% (*R*)-Perillyl Alcohol ((*R*)-**2**) and (1*RS*,5*R*)-5-Isopropyl-2-methylcyclohex-2-en-1-ol ((1*RS*,5*R*)-**4**)

A sample containing 44% (*R*)-**2** (152 mg) dissolved in acetone (1 mL, 5% v/v of total reaction volume) was mixed with ADH-hT cell-free extract (0.3 g_CWW_/mL, 19.3 U/mL, 6 mL, 116 U, 30% v/v of total reaction volume,) and pH 8.0 NaP_i_ buffer (50 mM to a total volume of 20 mL) with 420 μM NAD^+^ in a screw-capped glass bottle and incubated in an orbital mixer (180 rpm, 30 °C) for 6 h. The reaction mixture was extracted with EtOAc, dried (Na_2_SO_4_), and concentrated under reduced pressure to give a residue (138 mg) containing 30.4% (*R*)-**1** (NMR analysis). Two batches of the oxidation were combined and submitted to further purification.

### 2.6 Isolation of (*R*)-Perillaldehyde from the Crude Product Recovered from ADH-Mediated Oxidation

Sodium hydrogen sulfite (1.10 mmol, 114 mg) was added to a solution of the crude product (279 mg, 30% perillaldehyde, 0.55 mmol) recovered from the oxidation mediated by ADH-HT in 10:1 EtOH/H_2_O (2.2 mL). After the mixture was stirred for 30 min at room temperature, a white solid separated from the solution. Stirring was continued for 2 h, and then the solid was recovered by filtration, washed with EtOAc, and dried under vacuum at room temperature. The crystalline solid was dispersed in a 1:1 EtOAc/water (2 mL). Sodium carbonate (3 equiv) was added, and the mixture was stirred for 30 min. The organic phase was separated, washed with brine, dried (Na_2_SO_4_), and concentrated under reduced pressure to give perillaldehyde (66 mg, 22% isolated yield).

### 2.7 ADH-Mediated Oxidation of (*R*)-Perillyl Alcohol and Isolation of the Corresponding (*R*)-Perillaldehyde

A solution of (*R*)-**2** (152 mg, 1 mmol, 99% purity by NMR analysis) in acetone (1 mL, 5% v/v of total reaction volume) was mixed with ADH-hT CFE (0.3 g_CWW_/mL, 19.3 U/mL, 6 mL, 116 U, 30% v/v of total reaction volume) and pH 8.0 NaP_i_ buffer (50 mM, to a total volume of 20 mL) with 420 μM NAD^+^ in a screw-capped glass bottle and incubated in an orbital mixer (180 rpm, 30 °C) for 6 h. The reaction mixture was extracted with EtOAc, dried (Na_2_SO_4_), and concentrated under reduced pressure to give a residue (145 mg) containing 78% aldehyde **1** (GC/MS analysis).

Three batches of this reaction (420 mg) were combined and submitted to bulb-to-bulb distillation (10 mmHg, 109–111 °C) to give (*R*)-perillaldehyde (315 mg, 70% yield). ^**1**^ **H NMR** (400 MHz, CDCl_3_, ppm) δ: 9.43 (1H, s, C*H*O), 6.85–6.77 (1H, m, C*H*=), 4.79–4.76 (1H, m, C*H*H=), 4.74–4.76 (1H, m, C*H*H=), 2.54–2.39 (2H, m, hydrogens of the cyclohexene ring), 2.30–2.04 (3H, m, hydrogen of the cyclohexene ring), 1.95–1.86 (1H, m, hydrogen of the cyclohexene ring), 1.75 (3H, q, *J* = 1.1 Hz, C*H*_*3*_) 1.50–1.38 (1H, m, hydrogen of the cyclohexene ring). ^**13**^ **C NMR** (101 MHz, CDCl_3_, ppm) δ: 194.08, 150.80, 148.47, 141.41, 109.66, 40.83, 31.87, 26.48, 21.70, 20.82. **GC/MS** (EI): (*R*)-**1**: *t*_r_ = 10.43 min, *m*/*z* (%) = 150 (M^+^, 27), 135 (46), 107 (69), 79 (100). 98% ee by GC analysis using a column with a chiral stationary phase: (*S*)-**1**: *t*_r_ = 33.03 min, (*R*)-**1**: *t*_r_ = 33.88 min; [α]_D_ = +126 (*c* 1.0, CHCl_3_), lit. ref 7 [α]_D_ = +128.8 (CHCl_3_).

## 3 Results and Discussion

### 3.1 Base-Promoted Rearrangement of (4*R*)-Limonene Oxides

We started our investigation by considering an accidental discovery made by Eschinasi in 1973 when performing studies on the conversion of epoxy alcohols into the corresponding aluminum alcoholates by reaction with aluminum isopropylate.^29^ A catalytic amount (5–10 wt %) of Al­(O-*i*-Pr)_3_ promotes the easy rearrangement of oxirane rings to the corresponding allylic alcohols, even under solvent-free conditions. In the case of (4*R*)-limonene oxides, Eschinasi observed that the rearrangement occurred with preferential deprotonation at the C(7)-methyl rather than at the C(6)-methylene group, affording the final mixture reported in Table 1.

**Table 1:**
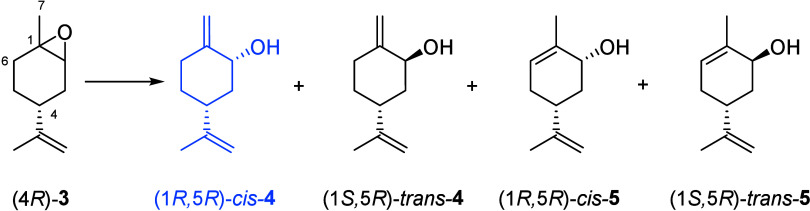
Rearrangement of Limonene Oxides with Catalytic Al­(O-*i*-Pr)_3_: Percentage Composition of the Reaction Mixture^a^

	composition (mol %)					
ref	*trans*-**3**	*cis*-**4**	*trans*-**4**	*cis*-**5**	*trans*-**5**	carvone
29	–	56	20	9	15	–
this work	8	44	22	7	16	3

a Percentage composition was determined by GC-FID analysis for the data from ref 29 and by ^1^H NMR analysis for our data. The blue color is used to highlight the effective precursor of the final perillaldehyde.

In our hands, the reaction performed on commercial limonene oxides (*trans*/*cis* 56:44 by NMR analysis) with Al­(O-*i*-Pr)_3_ (6.8 wt %) in refluxing toluene afforded the following mixture (molar composition by ^1^H NMR analysis, average of three reactions): 8% (4*R*)-*trans*-**3**, 44% (1*R*,5*R*)-**4**, 22% (1*S*,5*R*)-**4**, 7% (1*R*,5*R*)-**5**, 16% (1*S*,5*R*)-**5**, and 3% carvone (Table 1). The use of Al­(O-*i*-Pr)_3_ is very effective, and it can be employed in catalytic quantity. It is available commercially as a rather stable white solid that is widely used as a mild reagent for Meerwein–Ponndorf–Verley reduction,^30^ and it does not require inert atmosphere for usage, only moisture avoidance. It finds applications as a dehydrating agent, a viscosity regulator for varnishes, an intermediate for pharmaceuticals, and an antiperspirant in cosmetics.^31^ All attempts to isolate alcohols **4** from the reaction mixture in the enriched form by column chromatography failed.

### 3.2 Allylic Rearrangement by S_N_2′ Displacement of the Mesylate Derivatives of Alcohols **4** and **5**

The crude mixture recovered from the previous step was submitted to the allylic rearrangement of the corresponding mesylates according to ref 21 (Scheme 2b). After treatment with mesyl chloride and Et_3_N in dichloromethane, the quenching with saturated aqueous NaHCO_3_ was not as successful as that described in the paper. A complex mixture was obtained, in which perillyl alcohol was however identified by GC/MS analysis. We decided to investigate this reaction using the experimental conditions described in a recent paper for the same type of isomerization applied to a cannabidiol derivative. The authors suggested isolating the intermediate mesylate and carrying out the reaction with saturated aqueous NaHCO_3_ in DMF at 60 °C.^32^ Under these conditions we obtained a final mixture having the composition shown in Table 2 (entry “two steps”) as determined by ^1^H NMR.

**Table 2:**
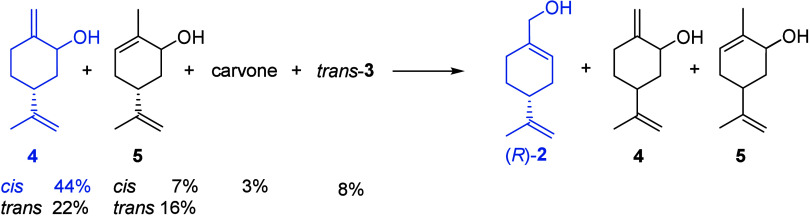
Allylic Rearrangement of Mesylate Derivatives: Percentage Molar Composition of the Reaction Mixture[Table-fn t2fn0]

	composition (mol %)			
reaction conditions	(*R*)-**2**	*cis*-**4**, *trans*-**4**	*cis*-**5**, *trans*-**5**	carvone
two steps^a^	41	5, 27	2, 23	2
one step^b^	40	5, 28	1, 22	4
after distillation^c^	44	4, 22	2, 25	3

a Percentage composition was determined by ^1^H NMR analysis. The blue color is used to highlight the effective precursor of final perillaldehyde.

b (i) MsCl (1.3 equiv) and Et_3_N (2 equiv) in DCM at 0 °C, then at r.t. for 4 h, followed by isolation of the reaction crude mixture; (ii) sat. aq NaHCO_3_ (1.5 equiv), DMF, 60 °C, 5 h.

c (i) MsCl (1.3 equiv) and Et_3_N (2 equiv) in acetone at 0 °C, then r.t. for 4 h; (ii) sat. aq NaHCO_3_ (1.5 equiv), room temperature, 5 h.

d Bulb-to-bulb distillation of the mixture recovered from the one-step reaction.

However, with the aim of avoiding solvents that pose great health risks and have high disposal costs, such as DCM and DMF, we further studied the mesylation of the mixture recovered from the Al­(O-*i*-Pr)_3_ reaction and the subsequent rearrangement using acetone as a solvent, without isolation of the intermediate mesylate. The following procedure was employed. To a solution of the crude mixture containing 66% derivatives **4** (NMR analysis) in acetone, Et_3_N (2 equiv) and mesyl chloride (1.3 equiv) were added dropwise at 0 °C. After stirring at room temperature until complete conversion of the alcohols into the corresponding mesylates (4 h), a saturated aqueous solution of NaHCO_3_ (1.5 equiv) was added. The mixture was stirred for 5 h and then extracted with 2-methyl-THF. Distillation of the solvent under reduced pressure left a residue with a composition (Table 2, entry “one step”) very similar to the one obtained by preparing the mesylate in DCM and running the subsequent rearrangement in DMF.

The analysis of the molar composition of the S_N_2′ allylic nucleophilic substitution highlighted that the mesylate of *trans*-**4** did not undergo the rearrangement but delivered the starting alcohol *trans*-**4** by simple hydrolysis of the mesylate. We hypothesize that even the low yields described for the S_N_2′ allylic nucleophilic substitution and isolation of perillyl alcohol in refs 20 and 21 (43% from the acetate derivative of **4** and 44% from **4**, respectively) are likely due to the lack of reactivity of *trans*-**4**. The molar percentage of *cis*-**4** obtained by the lithium diisopropylamide treatment of limonene oxides was approximately 65% (^1^H NMR), as described by Kamat et al.,^33^ to which both papers refer for the experimental conditions of the epoxide rearrangement.

The crude residue was submitted to bulb-to-bulb distillation to afford a sample containing 44% (*R*)-**2** (Table 2, entry “after distillation”). The enzymatic oxidation of perillyl alcohol was then considered as an alternative to classical chemical oxidation.

### 3.3 Enzymatic Oxidation of Perillyl Alcohol ((*R*)-**2**)

To the best of our knowledge, only a few studies describe the enzymatic oxidation of perillyl alcohol to perillaldehyde. Sato-Matsumoto and Ito^13^ isolated and expressed in *E. coli* two types of alcohol dehydrogenases, one from *Perilla frutescens* (PfAKR, an aldo-keto reductase) and one from *Perilla citriodora* (PcGeDH, a geraniol dehydrogenase). Both enzymes were able to oxidize perillyl alcohol (stereochemistry not defined in the text), although only on a screening scale (0.1 mM substrate concentration), with low conversion and the concomitant formation of the byproduct *trans*-shisool in the case of PcGeDH. In a subsequent work,^14^ Fujiwara and Ito isolated and expressed in *Saccharomyces cerevisiae* a P450 enzyme from *P. frutescens*; the enzyme was able to perform the oxidation of limonene (stereochemistry not defined in the text) to perillyl alcohol and then to perillaldehyde, but the production was extremely low, and perillyl alcohol and *trans*-shisool accumulated instead. Starting from perillyl alcohol, the conversion increased but was still unsatisfactory. As stated by the authors, the perillaldehyde synthesis pathway in *P. frutescens* remains unclear. In another work,^15^ Turner and his group performed directed evolution on a choline oxidase to improve its specificity and stability. A panel of 50 primary alcohols was screened, and the conversion of 10 mM perillyl alcohol was increased from 0 to almost 80% compared with the wild-type enzyme.

For our work, we decided to focus on alcohol dehydrogenases (ADHs, E.C. 1.1.1.1), as they can both catalyze the reaction of choice and regenerate the NAD^+^ cofactor by reducing a sacrificial substrate, i.e., acetone. Additionally, they do not require the use of other enzymes, such as peroxidases and catalases, to carry out their oxidative activity. An initial screening on the mixture recovered from allylic rearrangement (containing perillyl alcohol together with residual secondary alcohols **4** and **5**) was performed with the recombinant ADHs available in the enzyme collections of our research group (Table S1) under the following conditions: 50 mM NaP_i_ buffer (pH 7.0), 1% v/v acetone as both a cosolvent and the sacrificial substrate for NAD^+^ regeneration operated by the same ADH cell-free extract, 5 mM substrate concentration, and 24 h reaction time (Table S2). We found that the one from *Geobacillus stearothermophilus* (designated ADH-hT)^34^ could quantitively oxidize primary alcohol **2**, leaving completely unreacted both residual alcohols **4** and carveols **5**, and we decided to consider the possibility to take advantage of this chemoselectivity. Thus, two approaches to aldehyde (*R*)-**1** were investigated:

(A) chemoselective oxidation of alcohol **2** contained in the mixture (44% by NMR analysis) recovered from mesylate displacement by means of ADH-hT and subsequent isolation of the corresponding aldehyde by formation of the Bertagnini adduct;
(B) recovery of alcohol **2** from the reaction mixture by column chromatography, subsequent oxidation to (*R*)-**1**, and purification by distillation under reduced pressure.

#### 3.3.1 (A) Enzymatic Oxidation of the Mixture Containing 44% Perillyl Alcohol

We first studied the oxidation of the mixture containing 44% perillyl alcohol in addition to secondary alcohols **4** and **5** to optimize the reaction conditions and control the maintenance of chemoselectivity. The optimal conditions found for the mixture were then extended to perillyl alcohol oxidation. We performed a few reactions and a literature search to find out which parameters could be the most influential. In our hands, the volume percentage of acetone, the volume percentage of ADH-hT CFE (0.17 g_CWW_/mL, 10.9 U/mL), and the NAD^+^ concentration proved to be crucial. Additionally, the biochemical characterization of the enzyme performed by Bartolucci and his group^34^ highlighted the importance of a reaction temperature up to 50 °C (since the enzyme comes from a thermophilic bacterium) and a slightly alkaline pH for favoring the oxidizing activity of ADH-hT. These five variables were therefore selected for a Design of Experiments (DoE) approach aimed at maximizing the conversion and the yield toward perillaldehyde production. The minimum and maximum values considered for the variables were based on experimental and literature evidence (Table 3).

**Table 3:** List of Variables Considered for the DoE Study and the Corresponding Lower and Upper Limit Values^a^

factor code	variable	unit of measurement	lower limit	upper limit
A	acetone volume	% v/v	5	15
B	ADH-hT volume (units of enzyme)	% v/v (U)	15 (1.63)	30 (3.27)
C	[NAD^+^]	μM	250	500
D	pH	–	7	8
E	*T*	°C	30	50

a ADH-hT CFE (10.9 U/mL) was employed.

A five-variable half-factorial design elaborated through Design-Expert 13 was selected, consisting of 2^5–1^ experiments plus three central points for a total of 19 experiments, with a replicate for each point to increase the precision of the model (Table S3). Each experiment was performed in a 1 mL volume with a 50 mM concentration of the mixture (7.6 mg) containing 44% (*R*)-**2**, 50 mM NaP_i_ as a buffer, and ADH-hT as a cell-free extract (0.17 g_CWW_/mL, 10.9 U/mL). The comparison between the percentage molar composition of the starting mixture determined by ^1^H NMR and the percentage distribution obtained by GC/MS highlighted an underestimation of the perillyl alcohol content by GC/MS analysis (37% vs 44%). Nevertheless, for the purposes of this screening study, GC/MS was chosen for simplicity and rapidity. The enzyme provided both conversion of the substrate to (*R*)-**1** and regeneration of the NAD^+^ cofactor by reduction of acetone. After 24 h, each reaction mixture was extracted with EtOAc and analyzed by GC/MS. Two responses were studied: (a) conversion, calculated as the ratio between the peak area of perillaldehyde and the sum of the peak areas of perillaldehyde and perillyl alcohol, and (b) perillaldehyde yield, calculated as the ratio between the peak area of perillaldehyde and the sum of the peak areas of all the components of the mixture. It is worth noting that the formation of traces of perillic acid was also observed, but since it remained below 3% throughout the screening experiments it was not taken into consideration.

The ANOVA statistical analysis showed that the model obtained for relating the responses and the investigated parameters is significant, with *p* < 0.0001, and lack of fit is not significant relative to the pure error. However, the curvature check performed by the software based on the analysis of the central points highlighted a significant curvature in the model (Table S4), and an augmentation was suggested. A response surface methodology (RSM) optimal design was then applied by adding 10 experiments inside the same design space to check whether the curvature is in a desirable direction (Table S3).

The results of this study showed that the variable with the highest impact is the acetone percentage (Figure 2). When 15% v/v acetone was used, all of the reactions were characterized by poor conversion (0–10%), probably because of enzyme inactivation, while the lowest amount of acetone explored in the study (5% v/v) led to the highest conversion and yield. The volume of ADH-hT CFE influences positively both the conversion and yield, with a maximum reached with 30% v/v CFE; such high amount of catalyst required could be due to the toxicity of acetone and perillaldehyde or to a low expression level during protein production. Increasing temperature showed a negative effect on both conversion and perillaldehyde yield, despite the biochemical characterization of the enzyme from the literature showing a 3-fold increase in enzyme activity from 30 to 50 °C. This result can probably be ascribed to the faster inactivation of the enzyme at higher temperature. Interestingly, the negative effect of temperature is more impactful on perillaldehyde yield (Figure 2b), very likely due to partial leakage of the compound by evaporation, given its high volatility. The concentration of the cofactor NAD^+^ showed a positive effect on conversion up to a concentration of 420 μM; no improvement was observed with higher concentration. Finally, despite evidence from the literature, the pH value was almost irrelevant in the range tested. When the reaction was performed both at pH 7.0 and 8.0 with the other parameters at their optimal values, the use of pH 8.0 showed a 3% higher conversion and was therefore selected. The final optimized conditions were the following: 50 mM starting material, 5% v/v acetone, 30% v/v ADH-hT CFE (0.17 g_CWW_/mL, 10.9 U/mL), 420 μM NAD^+^, pH 8.0, and 30 °C, leading to 89.5% conversion in 24 h with final perillaldehyde content of 29% in the reaction mixture.

**Figure 2:**
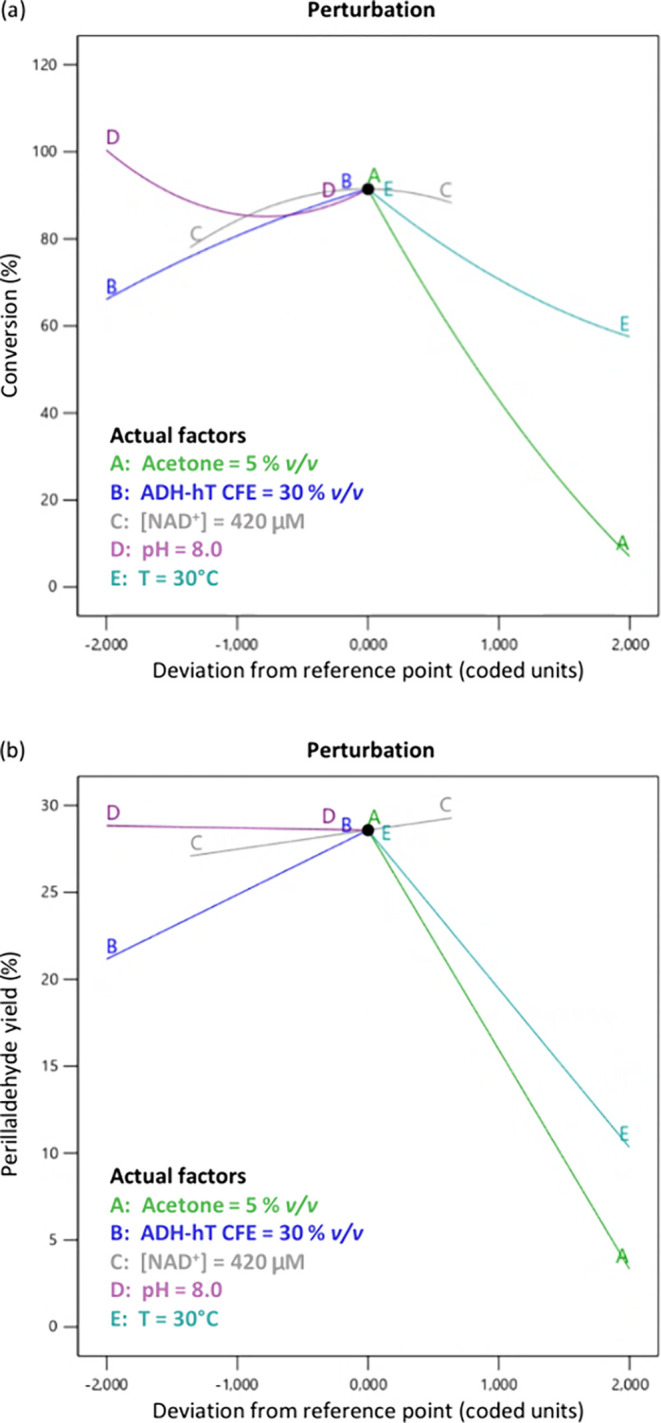
Perturbation plots showing the dependence of (a) conversion and (b) perillaldehyde yield on the values of the chosen parameters expressed in coded units (see Table 3 for the upper and lower limits of each parameter and Table S3 for the experimental data). The best values for all of the chosen parameters are listed inside the box of each plot.

Since the DoE investigation had shown that a higher quantity of enzyme was beneficial for the reaction, a more concentrated ADH-hT CFE (0.3 g_CWW_/mL, 19.3 U/mL) was employed (at 30% v/v) to double the amount of enzyme in an attempt to achieve high conversion in a shorter reaction time. The amount of the starting mixture was increased to 76 mg and the reaction volume to 10 mL to investigate the scalability of the oxidation. The time course of the reaction is reported in Figure 3 (see the data in Table S5). Under these conditions the conversion of alcohol (*R*)-**2** into aldehyde (*R*)-**1** reached 86% already after 6 h, but a prolonged reaction time led to a slight loss of aldehyde due to further oxidation to perillic acid.

**Figure 3:**
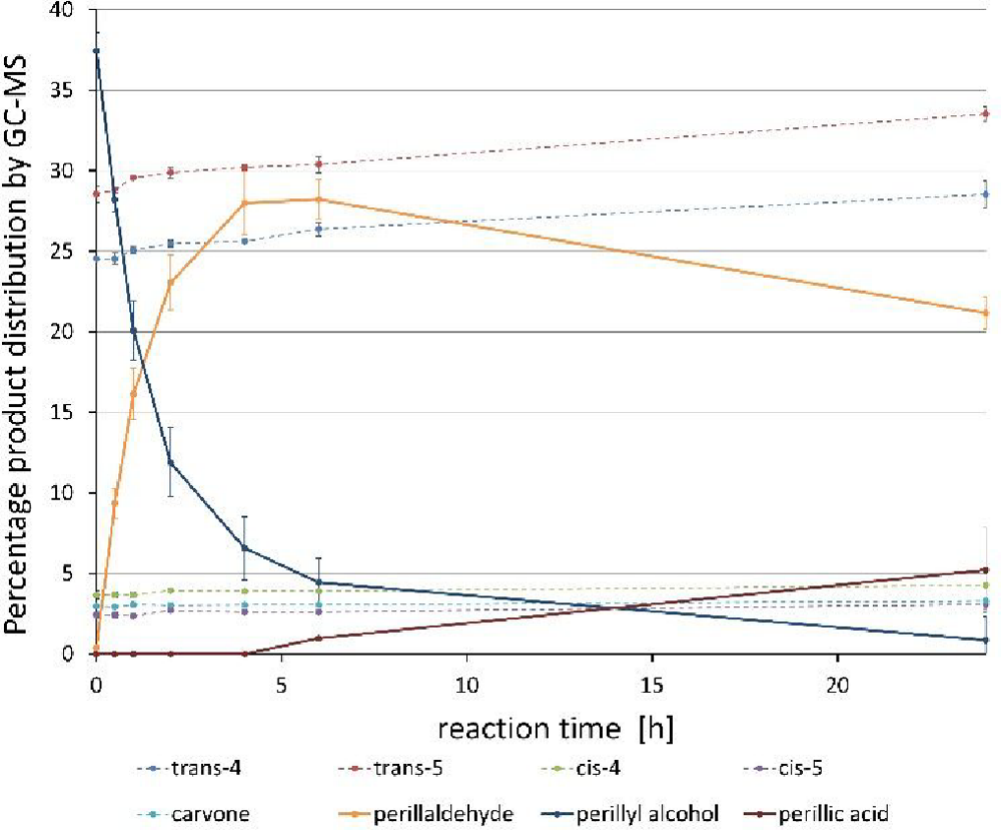
Time-course of the oxidation reaction of the mixture of perillyl alcohol ((*R*)-**2**). Reaction samples of 200 μL were taken after 0, 0.5, 1, 2, 4, 6, and 24 h. Each sample was extracted with 500 μL of EtOAc, centrifuged at 12,000 rpm for 2 min, and dried over Na_2_SO_4_, and the organic layer was transferred to a 500 μL vial for GC/MS. The reaction was performed in triplicate; error bars are calculated as standard deviation from the average of triplicates.

The oxidation of the mixture containing 44% perillyl alcohol was scaled-up (152 mg in 20 mL total volume, perillyl alcohol concentration 22 mM) under the optimized conditions with ADH-hT CFE (0.3 g_CWW_/mL, 19.3 U/mL) in a glass vial with a screw cap to avoid any loss of products due to their inherent volatility. After 6 h, the recovery of the reaction mixture by extraction with EtOAc was satisfactory (93%), and the following percentage molar composition was determined by ^1^H NMR analysis: 30.4% aldehyde (*R*)-**1**, 8.0% alcohol (*R*)-**2**, 2.6% perillic acid, 26.6% alcohols **4**, 28.8% carveols **5**, and 3.1% carvone. This composition corresponds to a conversion of perillyl alcohol to 74% perillaldehyde and 6% perillic acid.

The ability of aldehydes to promptly form the so-called Bertagnini adduct by reaction with an equimolar amount of sodium bisulfite was exploited for the isolation and purification of aldehyde (*R*)-**1** from the oxidation mixture. The reaction was carried out in 10:1 EtOH/H_2_O; the solid bisulfite adduct was recovered by filtration, and the aldehyde was regenerated by treatment with an aqueous solution of Na_2_CO_3_ in a biphasic mixture (EtOAc). Perillaldehyde was recovered as a pure product in 22% final isolated yield (Scheme 3, route (A)), starting from 44% perillyl alcohol. The enantiomeric excess of (*R*)-**1** (98% ee) was determined by GC analysis on a chromatographic column with a chiral stationary phase.

**Scheme 3:**
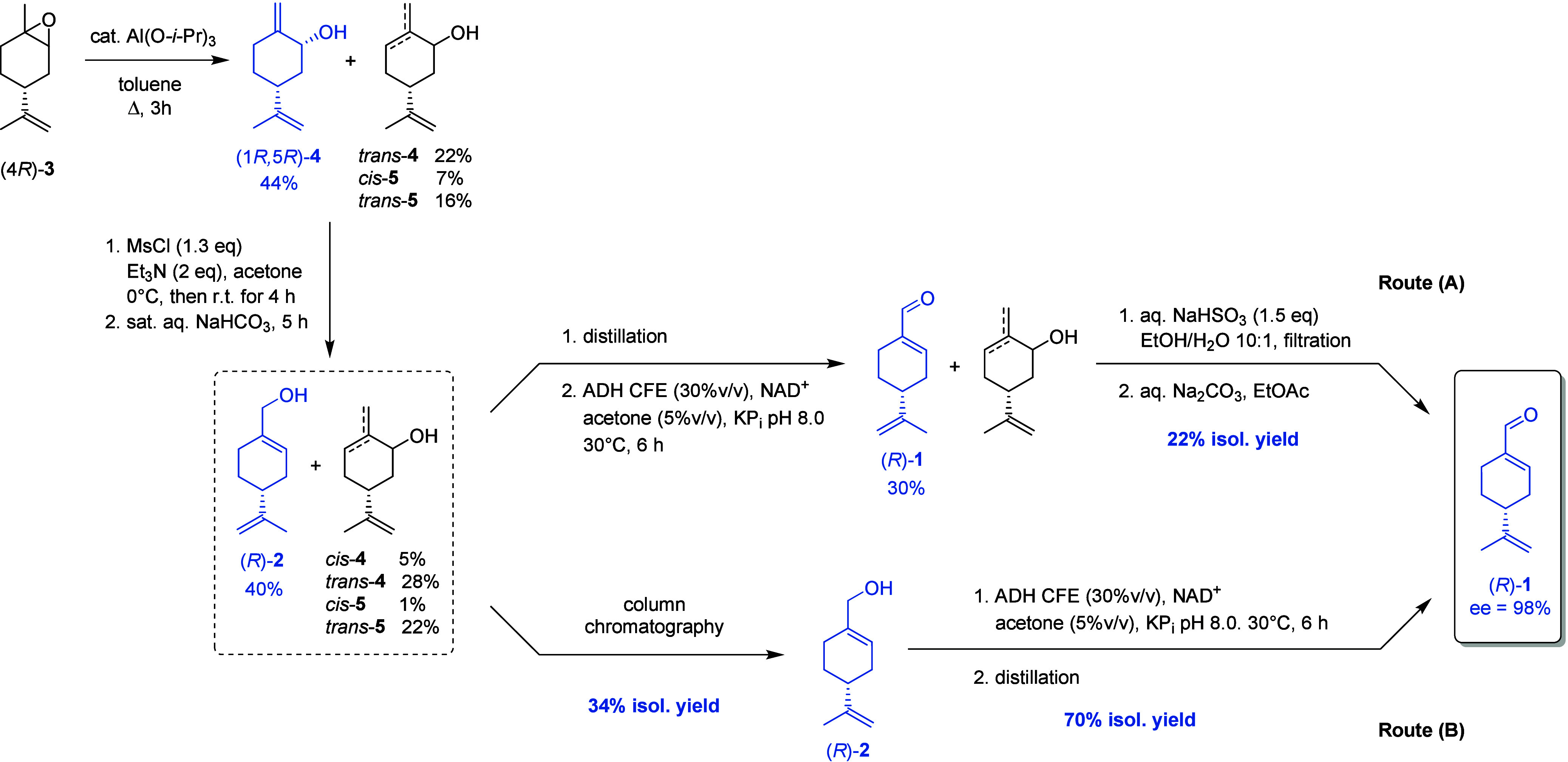
Synthetic Routes to (*R*)-**2** and (*R*)-**1** Described in This Work

#### 3.3.2 (B) Enzymatic Oxidation of (*R*)-Perillyl Alcohol

The optimized conditions found for the oxidation of 44% perillyl alcohol were also applied to the oxidation of (*R*)-**2** isolated from the mixture recovered after allylic rearrangement by column chromatography (isolated yield 34%). A few trials were carried out to set the right amount of enzyme, starting from the reaction conditions employed for the oxidation of 152 mg of the mixture with 44% (*R*)-**2** (perillyl alcohol concentration 22 mM). (*R*)-**2** (7.6 mg) in 1 mL total volume (perillyl alcohol concentration 50 mM) with 5% v/v acetone were treated with 30% v/v ADH-hT CFE (0.3 g_CWW_/mL, 19.3 U/mL) and 420 μM NAD^+^ at 30 °C and pH 8.0 for 6 h. A mixture containing 55% aldehyde **1**, 40% alcohol **2**, and 5% perillic acid was obtained, a very good result in spite of the higher substrate to enzyme ratio. By prolonging the reaction for 24 h, a higher concentration of perillaldehyde (75%) was obtained with a still negligible amount of perillic acid (6%), achieving a result similar to that observed in the oxidation of the mixture. The oxidation performed on 152 mg of (*R*)-**2** for 24 h afforded a final crude residue containing 78% (*R*)-**1**, 7% acid, and 15% residual alcohol (Scheme 3, route (B)). When three batches of the same reaction were combined, (*R*)-**1** could be isolated in a pure state with 98% ee by distillation under reduced pressure in 70% isolated yield.

### 3.4 Evaluation of Process Efficiency and Green Metrics

The evaluation of the efficiency and sustainability of the two processes described herein is based on the discussion of the following aspects: (i) choice of reagents, (ii) operative reaction conditions (reaction time, material usage, waste production), and (iii) overall impact of workup and purification procedures.

The green metrics parameters (yield, simplified environmental factor (sEF), atom economy (AE), and reaction mass efficiency (RME)) for our synthetic routes to alcohol (*R*)-**2** and aldehyde (*R*)-**1** (Scheme 3) and for those described in the literature were calculated according to ref 35 and are reported in Tables 4 and 5 for both the single steps and the overall sequence. The values were calculated under the assumption of complete recycling of reaction and postreaction solvents and water (see the Supporting Information for detailed calculations). The amounts of additives for the reaction workup were not described in the procedures reported in the literature and were therefore also not considered for the procedure described in this work. The EcoScale parameters^36^ of each step of the synthetic sequences compared herein were also calculated to quantitatively assess the impact due to yield, reagent price, safety, technical setup, reaction temperature and time, and postreaction operations.

**Table 4:** Green Metrics and EcoScale Parameters Calculated for Process (B) Described in This Work to Prepare Alcohol (*R*)-**2** and Those Reported in References 20 and 21

	ref 20	ref 21	this work
Full Process to Perillyl Alcohol			
overall yield	38.8	38.3	31.8
sEF	13.6	13.6	13.8
AE	0.19	0.23	0.34
RME	0.0836	0.0725	0.0764
Limonene Oxide Rearrangement			
yield	60.5	56.6	41.8
sEF	3.18	3.47	1.55
AE	0.43	0.43	1
RME	0.239	0.224	0.392
EcoScale	16.2	27.3	44.9
Allylic Rearrangement of Mesylate Derivatives			
yield	41.7	44.0	33.5
sEF	8.67	8.46	11.7
AE	0.32	0.34	0.336
RME	0.128	0.106	0.0853
EcoScale	–5.1	25.0	23.9

**Table 5:** Green Metrics and EcoScale Parameters Calculated for Process (B) Described in This Work to Prepare Aldehyde (*R*)-**1** and That Reported in Reference 7; For the Last Step of Oxidation to Perillaldehyde, the Green Metrics and EcoScale Parameters Calculated for Some Representative Known Chemical Oxidations of Perillyl Alcohol Are Also Reported

Full Process to Perillaldehyde		
parameter	ref 7	this work
overall yield	34.2	22.3
sEF	51.4	24.4
AE	0.11	0.29
RME	0.0194	0.0434

Limonene Oxide Rearrangement		
parameter	ref 7	this work
yield	56.9	41.8
sEF	15.8	1.55
AE	0.32	1
RME	0.0596	0.392
EcoScale	13.4	44.9

Allylic Rearrangement		
parameter	ref 7	this work
yield	61.0	33.5
sEF	3.47	11.7
AE	0.65	0.34
RME	0.289	0.0853
EcoScale	55.0	23.9

Oxidation to Perillaldehyde					
parameter	ref 7 ^a^	this work	ref 10 ^b^	ref 11 ^c^	ref 12 ^d^
yield	64	70.1	64	90.0	90.0
sEF	18.1	4.55	14.2	0.145	0.140
AE	0.16	0.71	0.63	0.82	0.82
RME	0.0524	0.180	0.066	0.87	0.88
EcoScale	11.0	66.0	38.0	49.0	51.0

a For the procedure reported in ref 7, the Pummerer rearrangement and the treatment with aqueous HgCl_2_ represent the final oxidation step.

b MnO_2_ (15 equiv) in hexane at r.t. for 2 h, filtration on Celite cake, and column chromatography.

c TEMPO (0.05 equiv) and CuCl (0.05 equiv) under O_2_ in bmimPF_6_ at 65 °C for 16 h, then the usual workup and column chromatography.

d Fe­(NO_3_)_3_ (10 mol %), TEMPO (10 mol %), and NaCl (10 mol %) under O_2_ in 1,2-dichloroethane at r.t. for 9 h, then the usual workup and column chromatography.

A preliminary comparison between our routes (A) and (B) led us to consider route (B) as more convenient than the other, taking into account the final yield of aldehyde **1**, the more effective use of the biocatalyst, and the ease of isolation of the final product (distillation vs formation of the Bertagnini adduct).

#### 3.4.1 Synthesis of Perillyl Alcohol ((*R*)-**2**)

Our synthetic route (B) to (*R*)-**2** is compared to those reported in refs 20 and 21 (Scheme 2, routes a and b, respectively). The values of the overall AE (0.19. 0.23, and 0.34 in Table 4) are almost identical since the reaction strategy adopted is the same in all of the processes, comprising the rearrangement of the epoxide ring of limonene oxide and the subsequent S_N_2′ allylic displacement of a suitable derivative.

##### Limonene Oxide Rearrangement

The parameters characterizing this first step (Table 4) were calculated considering that only *cis*-**4** underwent the subsequent allylic displacement in the compared processes. Thus, the actual yields for the formation of the *cis* diastereoisomer were used to obtain a more realistic evaluation of the procedures. In our approach, in spite of the lower selectivity toward alcohols **4**, obtained in a mixture with alcohols **5**, the use of a catalytic quantity of aluminum isopropylate in refluxing toluene solution in a common reactor setup (compared to the use of stoichiometric LDA at low temperature in dry solvent under an inert atmosphere) ensures higher atom economy, lower waste production, and more effective conversion of the starting materials into the final product in terms of mass. This is quantitatively shown by the values calculated for this specific step for AE (1 vs 0.43 for the other two processes), sEF (1.55 vs 3.18 and 3.47), and RME (0.392 vs 0.239 and 0.224) and by a more favorable EcoScale parameter (44.9 vs 16.2 and 27.3). The crude product was submitted directly to the next step, as column chromatography would not have allowed separation of the target compound from the byproducts.

##### Allylic Rearrangement of Mesylate Derivatives

With regard to the second step, the allylic S_N_2′ displacement of the mesylate promoted with aqueous NaHCO_3_ is more straightforward under the operational point of view than the rearrangement of the acetate derivative with palladium catalyst, as it appears from the negative EcoScale parameter of the latter (−5.1) in Table 4. This value reflects the safety issues posed by the use of multiple reagents and solvents in the three-step sequence to convert derivative **4** into perillyl alcohol **2** via the acetate intermediate. The lower content of *cis*-**4** in our reaction mixture has a negative effect on the sEF and RME values, but it is compensated by the good impact of the reaction methodology. The one-pot transformation of *cis*-**4** into (*R*)-**2** via the mesylate intermediate in acetone as an organic solvent is a more sustainable approach than the use of DCM for mesylation followed by treatment with aqueous NaHCO_3_ in DCM/water. The EcoScale parameter of our route, including column chromatography, is comparable to the one characterizing the synthesis of (*R*)-**2** described in ref 21 (23.9 vs 25.0). We performed column chromatography with an automated flash purification system to limit solvent use and reduce time: 3.5 g of mixture was separated on a silica gel cartridge (120 g) starting from 96:4 hexane/EtOAc to 6:4 hexane/EtOAc using a 2.3 L total volume of hexane/EtOAc mixture in 41 min.

We also chose 2-MeTHF for the workup of the reaction because it is a biodegradable solvent prepared from renewable feedstocks and it can be used to azeotropically dry the product before isolation, simplifying workup and avoiding drying salts such as Na_2_SO_4_.

#### 3.4.2 Synthesis of Perillaldehyde ((*R*)-**1**)

The overall yield for perillaldehyde synthesis described in ref 7 is higher than that obtained in this work, but the other green metrics (sEF, AE, and RME) and the EcoScale scores of each reaction step reported in Table 5 clearly show the advantages of our approach, in spite of the low selectivity of the first step.

The use of problematic stoichiometric reagents under anhydrous conditions under an inert atmosphere at very low temperature (−78 °C) is avoided. The reaction of the intermediate sulfoxide of ref 7 with trifluoroacetic anhydride followed by treatment with aqueous HgCl_2_ is to be compared to the ADH-mediated oxidation. The latter is very effective and exploits acetone as the sacrificial substrate, and the enzyme is itself biodegradable and can be produced by renewable sources. This oxidation step was also compared to other chemical oxidations described on perillyl alcohol in the literature (Table 5).

The least convenient oxidation method is the one characterized by the great excess of MnO_2_ and the lowest yield. The two strategies using molecular oxygen as an oxidant show good values of the green metrics, but they employ metal-based reagents (albeit in catalytic quantities) that need specific disposal and disadvantageous solvents: either a highly expensive ionic liquid or a chlorinated solvent. The EcoScale parameters clearly show the superiority of the enzyme-mediated oxidation. Most of the waste of the bio-oxidation is represented by water and biodegradable components of cell-free extract. Perillaldehyde can be easily recovered in a pure state by distillation under reduced pressure.

## 5 Conclusions

The synthetic approach to (*R*)-perillaldehyde has been developed by starting from known routes and progressively applying the principles of green chemistry. Limonene oxides, easily available from renewable limonene, are still employed as starting materials. The first step of epoxide rearrangement has been performed under catalytic conditions, avoiding the use of stoichiometric water- and air-sensitive reagents, which require specific experimental setup and glassware and operate at very low temperature. Al­(O-*i*-Pr)_3_ can be used in catalytic quantity and is more easily and safely handled, even though it promotes a less selective rearrangement of the oxirane ring (44% *cis*-**4** in the final mixture vs approximately 65% when *n*-BuLi is used). Work is in progress to develop a procedure for separating *cis*-**4** from *trans*-**5**, the two main components of the reaction mixture, in order to recover and use both of them. *trans*-**5** is the isomer of carveol which is not accessible by reduction of carvone with NaBH_4_ in DCM/MeOH, since the reaction affords *cis*-**5** with 81% de.^37^

For the allylic rearrangement by S_N_2′ displacement of the mesylate derivatives of alcohols **4** and **5**, we could obtain the same results in terms of composition of the final mixture by substituting DCM and DMF with acetone and aqueous acetone, respectively. The extraction of the final mixture with water-immiscible 2-MeTHF also made it possible to avoid drying of the organic solution with anhydrous Na_2_SO_4_, taking advantage of the formation of an azeotrope between this solvent and water. To date, no effective safer alternative to mesyl chloride has been identified for the derivatization of alcohol *cis*-**4** allowing for such a controlled rearrangement toward perillyl alcohol.

Finally, the development of the enzymatic oxidation of intermediate perillyl alcohol with the cell-free extract of ADH-hT and NAD^+^ regeneration by the ADH itself in the presence of acetone, used as both the sacrificial substrate and organic cosolvent, greatly improved the sustainability of the process. All of the side products of the enzymatic oxidation are easily disposed of and biodegradable.

With regard to the scalability of the process, we have already experimented with the reaction using catalytic Al­(O-*i*-Pr)_3_ and the allylic rearrangement of the intermediate mesylates on a 10 g scale, as it is herein described. These two steps can be quite easily scaled up, since they involve the handling of classic reagents and do not require specialized equipment. The production of ADH-hT certainly needs to be improved, in particular by applying the strategy to enhance enzymatic activity by removing *E. coli* basal proteins through high-temperature treatment, given the reported thermal stability of ADH-hT isolated from a thermophilic microorganism.^38^ Increasing biocatalyst production is generally a predictable and straightforward process if a well-established small-scale procedure has already been optimized. It is simply a matter of the equipment availability. The number of kilogram-scale processes based on the use of alcohol dehydrogenase is increasing,^39^ thus highlighting the consideration given to this strategy for the development of more sustainable manufacturing processes.

## Supplementary Material



## Notes

Experimental data for the ADH screening for perillyl alcohol oxidation, experimental data for the DoE study, experimental data for the time course of the ADH-hT-mediated oxidation of the mixture containing alcohol **2**, calculation of green metrics and EcoScale parameters, GC analyses for the determination of the ee of (*R*)-**1**, and ^1^H and ^13^C NMR spectra (PDF)
