# Supplementary material for: Chemo-enzymatic Approach to (R)‑Perillaldehyde: Improving the Sustainability of the Reaction Steps with the Principles of Green Chemistry
Source: Org Process Res Dev. 2025 Nov 24;30(1):77–87. doi: 10.1021/acs.oprd.5c00340 (PMC12817304; doi:10.1021/acs.oprd.5c00340)

Chemo-enzymatic approach to (*R*)-perillaldehyde: improving the sustainability of the reaction steps with the principles of Green Chemistry

Federico Acciaretti,[a] Celeste Nobbio,[a] Natale Crisafulli,[a] Martina Arosio,[a] Francesco G. Gatti,[a] Fabio Parmeggiani,[a] Elisabetta Brenna*[a]

Dipartimento di Chimica, Materiali ed Ingegneria Chimica “Giulio Natta”, Politecnico di Milano, Piazza Leonardo da Vinci 32, 20133, Milano, Italy.
E-mail: mariaelisabetta.brenna@polimi.it; phone: +39 02 23993077

Supporting Information

Table S1. List of alcohol dehydrogenases screened to find the suitable catalyst for perillyl alcohol oxidation.

ADH	Microrganism	Plasmid	Literature reference
ReSADH	*Rhodococcus erythropolis* DSM 43297	pPB01	K. Abokitse and W. Hummel, *Appl. Microbiol. Biotechnol.*, 2003, **62**, 380-386.
SyADH	*Sphingobium yanoikuyae*	pET24b	W. Kroutil *et al.*, *Org. Lett.* 2008, **10**, 2155–2158.
ADH-A	*Rhodococcus ruber* DSM 44541	pK470	M. Widersten *et al.*, *J. Mol. Catal. B Enzym.,* 2014, **99**, 68–78
ADH-hT	*Geobacillus stearothermophilus*	pK470	A. Guagliardi *et al.*, *Int. J. Biochem. Cell Biol.*, 1996, **28**, 239-246.

Amino acid sequences of the ADHs used

ReSADH

MKAIQYTRIGAEPELTEIPKPEPGPGEVLLEVTAAGVCHSDDFIMSLPEEQYTYGLPLTLGHEGAGKVAAVGE
GVEGLDIGTNVVVYGPWGCGNCWHCSQGLENYCSRAQELGINPPGLGAPGALAEFMIVDSPRHLVPIGDLDPV
KTVPLTDAGLTPYHAIKRSLPKLRGGSYAVVIGTGGLGHVAIQLLRHLSAATVIALDVSADKLELATKVGAHE
VVLSDKDAAENVRKITGSQGAALVLDFVGYQPTIDTAMAVAGVGSDVTIVGIGDGQAHAKVGFFQSPYEASVT
VPYWGARNELIELIDLAHAGIFDIAVETFSLDNGAEAYRRLAAGTLSGRAVVVPGL

SyADH

MTTLPTVLITGASSGIGATYAERFARRGHDLVLVARDKVRLDALAARLRDESGVAVEALQADLTRPADLAAVE
IRLREDARIGILINNAGMAQSGGFVQQTAEGIERLITLNTTALTRLAAAVAPRFVQSGTGAIVNIGSVVGFAP
EFGMSIYGATKAFVLFLSQGLNLELSPSGIYVQAVLPAATRTEIWGRAGIDVNTLPEVMEVDELVDAALVGFD
RRELVTIPPLHVAARWDALDGARQGLMSDIRQAQAADRYRPEA

ADH-A

MKAVQYTEIGSEPVVVDIPTPTPGPGEILLKVTAAGLCHSDIFVMDMPAAQYAYGLPLTLGHEGVGTVAELGE
GVTGFGVGDAVAVYGPWGCGACHACARGRENYCTRAADLGITPPGLGSPGSMAEYMIVDSARHLVPIGDLDPV
AAAPLTDAGLTPYHAISRVLPLLGPGSTAVVIGVGGLGHVGIQILRAVSAARVIAVDLDDDRLALACKVGADA
AVKSGAGAADAIRELTGGQGATAVFDFVGAQSTIDTAQQVVAVDGHISVVGIHAGAHAKVGFFMIPFGASVVT
PYWGTRSELMEVVALARAGRLDIHTETFTLDEGPAAYRRLREGSIRGRGVVVP

ADH-hT

MKAAVVEQFKEPLKIKEVEKPTISYGEVLVRIKACGVCHTDLHAAHGDWPVKPKLPLIPGHEGVGIVEEVGPG
VTHLKVGDRVGIPWLYSACGHCDYCLSGQETLCEHQKNAGYSVDGGYAEYCRAAADYVVKIPDNLSFEEAAPI
FCAGVTTYKALKVTGAKPGEWVAIYGIGGLGHVAVQYAKAMGLNVVAVDIGDEKLELAKELGADLVVNPLKED
AAKFMKEKVGGVHAAVVTAVSKPAFQSAYNSIRRGGACVLVGLPPEEMPIPIFDTVLNGIKIIGSIVGTRKDL
QEALQFAAEGKVKTIIEVQPLEKINEVFDRMLKGQINGRVVLTLEDK

Table S2. Preliminary results of ADH-mediated oxidations.

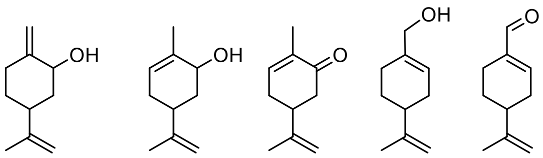

cis and trans-**4** *cis*- and *trans*-**5** carvone **2** **1**

ADH	*trans-4 (%)*	*trans-5 (%)*	cis-4 *(%)*	cis-5 *(%)*	carvone *(%)*	aldehyde 1 *(%)*	alcohol 2 v*(%)*
	18.8	26.8	3.8	2.9	3.4	0	44.3
ReSADH	25.5	17.9	3.5	2.5	17.7	22.7	10.2
SyADH	10.2	15.6	8.3	2.1	17.3	1.3	45.2
ADH-A	21.0	20.6	3.1	2.8	12.3	5.4	34.8
ADH-hT	20.6	27.9	3.9	4.3	3.0	37.4	2.9

Table S3. List of experiments performed for the screening investigation, with the specific combination of variable values and the obtained responses. Each block was performed on a different day. Block 3 is the augmentation of the model performed to adjust the curvature.

			Factor A	Factor B	Factor C	Factor D	Factor E	Response 1	Response 2
Std	Block	Run	Acetone % (v/v)	ADH-hT % (v/v)	[NAD$^+$] μM	pH	T °C	Conversion %	Perillaldehyde yield %
5	Block 1	1	5.0	30.0	250.0	7.0	30.0	78.2	27.5
17	Block 1	2	5.0	15.0	250.0	8.0	30.0	66.2	23.1
27	Block 1	3	15.0	15.0	500.0	8.0	30.0	4.9	2.0
19	Block 1	4	15.0	15.0	250.0	8.0	50.0	0.0	0.0
23	Block 1	5	15.0	30.0	250.0	8.0	30.0	5.1	2.0
25	Block 1	6	5.0	15.0	500.0	8.0	50.0	21.3	6.2
31	Block 1	7	15.0	30.0	500.0	8.0	50.0	0.0	0.0
13	Block 1	8	5.0	30.0	500.0	7.0	50.0	78.4	13.2
9	Block 1	9	5.0	15.0	500.0	7.0	30.0	67.8	24.3
21	Block 1	10	5.0	30.0	250.0	8.0	50.0	41.5	9.6
7	Block 1	11	15.0	30.0	250.0	7.0	50.0	0.0	0.0
35	Block 1	12	10.0	22.5	375.0	7.5	40.0	30.6	11.0
3	Block 1	13	15.0	15.0	250.0	7.0	30.0	3.0	1.2
34	Block 1	14	10.0	22.5	375.0	7.5	40.0	17.9	6.8
29	Block 1	15	5.0	30.0	500.0	8.0	30.0	89.7	27.9
15	Block 1	16	15.0	30.0	500.0	7.0	30.0	11.0	4.3
33	Block 1	17	10.0	22.5	375.0	7.5	40.0	17.5	6.6
11	Block 1	18	15.0	15.0	500.0	7.0	50.0	4.6	1.7
1	Block 1	19	5.0	15.0	250.0	7.0	50.0	12.2	4.2
16	Block 2	20	15.0	30.0	500.0	7.0	30.0	8.6	3.4
24	Block 2	21	15.0	30.0	250.0	8.0	30.0	4.2	1.7
6	Block 2	22	5.0	30.0	250.0	7.0	30.0	81.0	27.4
8	Block 2	23	15.0	30.0	250.0	7.0	50.0	0.0	0.0
26	Block 2	24	5.0	15.0	500.0	8.0	50.0	22.8	6.6
20	Block 2	25	15.0	15.0	250.0	8.0	50.0	0.0	0.0
37	Block 2	26	10.0	22.5	375.0	7.5	40.0	15.9	6.1
10	Block 2	27	5.0	15.0	500.0	7.0	30.0	71.1	25.5
36	Block 2	28	10.0	22.5	375.0	7.5	40.0	16.0	6.1
22	Block 2	29	5.0	30.0	250.0	8.0	50.0	42.7	9.7
18	Block 2	30	5.0	15.0	250.0	8.0	30.0	51.6	18.4
38	Block 2	31	10.0	22.5	375.0	7.5	40.0	12.3	4.8
30	Block 2	32	5.0	30.0	500.0	8.0	30.0	89.1	28.2
32	Block 2	33	15.0	30.0	500.0	8.0	50.0	0.9	0.4
2	Block 2	34	5.0	15.0	250.0	7.0	50.0	13.1	4.4
4	Block 2	35	15.0	15.0	250.0	7.0	30.0	3.0	1.2
12	Block 2	36	15.0	15.0	500.0	7.0	50.0	0.0	0.0
28	Block 2	37	15.0	15.0	500.0	8.0	30.0	2.3	0.9
14	Block 2	38	5.0	30.0	500.0	7.0	50.0	72.0	13.3
**RSM Optimal**									
45	Block 3	39	8.6	15.2	489.0	7.0	43.1	17.7	6.6
43	Block 3	40	5.0	30.0	373.6	7.6	37.0	66.4	22.7
46	Block 3	41	13.6	23.5	500.0	8.0	48.7	0.0	0.0
39	Block 3	42	5.0	15.0	250.0	7.0	30.0	49.3	9.9

41	Block 3	43	12.0	30.0	362.5	8.0	30.0	25.6	18.6
44	Block 3	44	8.6	15.2	489.0	7.0	43.1	19.1	7.1
42	Block 3	45	12.0	30.0	362.5	8.0	30.0	24.0	9.3
48	Block 3	46	5.0	15.0	250.0	8.0	50.0	10.2	3.7
47	Block 3	47	8.5	30.0	250.0	7.5	50.0	9.1	3.4
40	Block 3	48	15.0	15.0	250.0	8.0	30.0	2.0	0.8

Table S4. Curvature check performed by Design-Expert 13® software. The curvature of the model appears significant, therefore an augmentation was suggested.

Term	Adjusted F-value	Model p-value	
Model	137.91	< 0.0001	significant
Curvature	26.20	< 0.0001	significant
Lack of fit	0.7712	0.6966	

ADH-mediated oxidation of the mixture containing 44 % (*R*)-perillyl alcohol (NMR analysis): reaction variables optimisation by DoE

A five-variables half-factorial design elaborated through Design-Expert 13® was selected, consisting of 2^{5-1} experiments plus 3 central point for a total of 19 experiments, with a replicate for each point to increase the precision of the model (Table S3). Each experiment was performed in a 1 mL volume with a 50 mM concentration of the mixture (7.6 mg) containing 44 % perillyl alcohol (*R*)-**2** as substrate, 50 mM NaP_i as buffer, and ADH-hT as crude cell lysate (0.17 g_{CWW}/mL). The values of the five variables are reported in Table S3. After 24h the reaction was extracted with 500 µL EtOAc, centrifuged at 12000 rpm for 2 minutes, dried over Na_2SO_4, and the organic layer was transferred to a 500 µL vial for GC-MS analysis. Two responses were studied: (a) conversion, calculated as the ratio between the peak area of perillaldehyde and the sum of the peak areas of perillaldehyde and perillyl alcohol; (b) perillaldehyde yield, calculated as the ratio between the peak area of perillaldehyde and the sum of the peak areas of all the components of the mixture.

Table S5. Experimental data for studying the time-course of the ADH-hT – mediated oxidation of the mixture containing perillyl alcohol **2** (44% by NMR analysis, 37.5% by GC/MS analysis). Data are reported as mean values of the GC/MS percentages of three replicates and the corresponding standard deviations (σ).

Time (h)	*trans*-4	σ	*trans*-5	σ	*cis*-4	σ	*cis*-5	σ	carvone	σ	ald. 1	σ	alc. 2	σ	perillic acid	σ
0	24.55	0.08	28.55	0.49	3.65	0.24	2.43	0.20	2.97	0.02	0.39	0.67	37.5	1.1	0.0	0.0
0.5	24.55	0.35	28.81	0.17	3.69	0.17	2.43	0.19	2.95	0.10	9.36	0.95	28.2	0.7	0.0	0.0
1	25.08	0.23	29.58	0.07	3.67	0.16	2.37	0.03	3.07	0.02	16.14	1.61	20.1	1.8	0.0	0.0
2	25.49	0.22	29.88	0.34	3.94	0.12	2.70	0.08	3.04	0.05	23.06	1.67	11.9	2.1	0.0	0.0
4	25.63	0.06	30.23	0.16	3.90	0.08	2.61	0.08	3.06	0.06	28.00	1.96	6.6	2.0	0.0	0.3
6	26.38	0.43	30.39	0.50	3.91	0.06	2.62	0.08	3.08	0.05	28.22	1.23	4.4	1.5	1.0	0.2
24	28.55	0.86	33.55	0.45	4.28	0.07	3.09	0.29	3.30	0.03	21.17	1.01	0.9	1.5	5.2	2.6

ADH-mediated oxidation of the mixture containing 44 % (*R*)-perillyl alcohol (NMR analysis): study of the time course of the reaction

A sample (76 mg) containing 44 % (*R*)-**2** dissolved in acetone (500 µL, 5% v/v of total reaction volume) was mixed with ADH-hT cell lysate (0.30 $g_{WetCells}$/mL, 3 mL, 30 % v/v of total reaction volume) and NaP_i buffer pH = 8 (50 mM, to a total volume of 10 mL) with 420 µM NAD^+ in a screw-capped glass bottle and incubated in an orbital mixer (180 rpm, 30 °C). Reaction samples of 200 µL were taken after 0, 0.5, 1, 2, 4, 6, and 24 hours. Each sample was extracted with 500 µL EtOAc, centrifuged at 12000 rpm for 2 minutes, dried over Na_2SO_4, and the organic layer was transferred to a 500 µL vial for GC-MS. The reaction was performed in triplicate, error bars were calculated as standard deviation of the average of triplicates.

Calculation of the Green metrics and EcoScale parameters

The values of the simplified Environmental Factor (sE-Factor), Atom Economy (AE), Reaction Mass Efficiency (RME) for the procedures described in this work were calculated under the hypothesis of completely recycling reaction and post-reaction solvents and water, according to the formulas suggested by F. Roschangar, R. A. Sheldon, C. H. Senanayake *Green Chem.*, 2015, **17**, 752 – 768.

The EcoScale parameters were calculated according to K. Van Aken, L. Strekowski and L. Patiny, Beil. J. Org. Chem., 2006, 2, No. 3. doi:10.1186/1860-5397-2-3.

Synth. Commun. 1988, 18, 1905 - 1911 - I STEP				sE-Factor	RME	yield	AE
**Raw materials and reactants (g)**	**mmol**	**MM**	**eq**	15,8	0,0596	56,9	0,32
limonene oxides							
10,00	65,8	152,0					
cyclohexylisopropylamine							
37,18	263,2	141,3	4,0				
BuLi							
16,87	263,2	64,1	4,0				
MeMgBr							
31,38	263,2	119,2	4,0				
sum of raw materials, reactants, catalysts							
95,43							
**Products [g]**							
purity (%)							
**65,0**							
alcohol **4** (only *cis*-**4**)		152,00					
	**yield (%)**		mmol				
5,69	**87,5**		57,58				
sum of total waste			37,42				
89,75							
cyclohexylisopropylamine in toluene, BuLi in hexane , MeMgBr in Et_2O, limonene oxide in toluene, 0°C, 24 h, flash chromatography, specific apparatus for manipulating BuLi							

Synth. Commun. 1988, 18, 1905 – 1911-II STEP				sE-Factor	RME	yield	AE
**Raw materials and reactants (g)**	**mmol**	**MM**	**eq**	3,47	0,289	61,0	0,65
Alcohol **4** (cis+trans)							
8,75	57,6	152,0					
phenyl sulfenyl chloride							
8,33	57,6	144,6	1,0				
triethylamine							
14,57	143,9	101,2	2,5				
sum of raw materials, reactants, catalysts							
31,64							
**Products [g]**							
purity (%)							
perillyl phenyl sulfoxide	**yield (%)**						
9,13	**61,0**	260,0					
sum of total waste							
22,51							
dry DCM, -78°C, under nitrogen, flash chromatography							

Synth. Commun. 1988, 18, 1905 – 1911-III STEP				sE-Factor	RME	yield	AE
**Raw materials and reactants (g)**	**mmol**	**MM**	**eq**	18,1	0,0524	64,0	0,16
perillyl phenyl sulfoxide							
9,13	35,1	260,0					
2,6-lutidine							
11,29	105,4	107,2	3,0				
tifluoroacetic anhydride							
29,73	105,4	282,1	3,0				
HgCl2							
14,21	52,3	271,5	1,5				
sum of raw materials, reactants, catalysts							
64,36							
**Products [g]**							
purity (%)							
perillaldehyde	**yield (%)**						
3,37	**64,0**	150,0					
sum of total waste							
60,98							
Acetonitrile, - 40°C, then H_2O at 25°C, flash chromatography							

Synth. Commun. 1988, 18, 1905 – 1911	
**3 steps, overall yields =**	34,2
**sE-Factor=**	51,4
**AE=**	0,11
**RME=**	0,0194

TeLe 2014, 55, 1431-1433 - I STEP				sE-Factor	RME	yield	AE
**Raw materials and reactants (g)**	**mmol**	**MM**	**eq**	3,18	0,239	60,5	0,43
limonene oxides cis + trans							
0,261	1,71	152,0					
diisopropylamine							
0,266	1,89	141,3	1,1				
BuLi							
0,132	2,06	64,1	1,2				
sum of raw materials, reactants, catalysts							
0,659							
**Products [g]**							
purity (%)							
**65,0**							
alcohol **4** (only *cis* -**4**)	**yield (%)**	152,0	mmol				
0,158	**93,0**		1,59				
sum of total waste			1,04				
0,501							
THF, -78°C, distillation, specific apparatus for manipulating BuLi							

TeLe 2014, 55, 1431-1433 - II STEP				sE-Factor	RME	yield	AE
**Raw materials and reactants (g)**	**mmol**	**MM**	**eq**	1,72	0,367	63,1	0,58
alcohol **4** (65% cis+35% trans)							
0,242	1,59	152,0					
acetic anhydride							
0,163	1,59	102,0	1,0				
pyridine							
0,126	1,59	79,0	1,0				
sum of raw materials, reactants, catalysts							
0,531							
**Products [g]**							
purity (%)							
**65,0**							
acetate of **4** (only *cis* -**4** acetate)							
	**yield (%)**		mmol				
0,195	**97,0**	194,0	1,55				
sum of total waste			1,01				
0,336							
DCM as a solvent. The authors don't describe the conditions. They write: standard conditions.							

TeLe 2014, 55, 1431-1433 - III STEP				sE-Factor	RME	yield	AE
**Raw materials and reactants (g)**	**mmol**	**MM**	**eq**	1,14	0,466	49,4	1,00
acetate of **4** (cis+ trans)							
0,300	1,546	194,0					
**Pd(PPh3)4 cat.**							
0,018	0,015	1155,6					
sum of raw materials, reactants, catalysts							
0,318							
**Products [g]**							
purity (%)							
**65,0**							
perillyl acetate	**yield (%)**		mmol				
0,148	**76,0**	194,0	1,18				
sum of total waste			0,76				
0,170							
THF, 110°C in a sealed tube for 15 h, flash chromatography, impure of starting material (35% starting, 65% final product)							

TeLe 2014, 55, 1431-1433 - IV STEP				sE-Factor	RME	yield	AE
**Raw materials and reactants (g)**	**mmol**	**MM**	**eq**	8,67	0,128	41,7	0,32
perillyl acetate (purity 65%)				Calculated from step II			
0,228	1,18	194,0					
potassium carbonate							
0,244	1,76	138,2	1,5				
sum of raw materials, reactants, catalysts							
0,472							
**Products [g]**							
purity (%)							
perillyl alcohol	**yield (%)**		mol were calculated starting from 300 mg of acetate of compound **4** with 43% yield over two steps (1st step yiel = 76%, 2nd step yield = 56.6%)				
0,101	**56,6**	152,0	0,665				
sum of total waste							
0,371							
MeOH, room temperature, column chromatography							

TeLe 2014, 55, 1431-1433	
**4 steps, overall yields =**	38,8
**sE-Factor=**	13,6
**AE=**	0,1866
**RME=**	0,0836

Chem Eur J 2017, 23, 9761-9765 - I STEP				sE-Factor	RME	yield	AE
**Raw materials and reactants (g)**	**mmol**	**MM**	**eq**	3,47	0,224	56,6	0,43
limonene oxides (cis + trans)							
29,9	196,6	152,0					
diisopropylamine							
30,5	216,2	141,3	1,1				
BuLi							
15,1	235,9	64,1	1,2				
sum of raw materials, reactants, catalysts							
75,5							
**Products [g]**							
purity (%)							
65,0							
alcohol **4** (only cis-**4**)	**yield (%)**	152,0	mmol				
16,9	**87,0**		171,0				
sum of total waste			111,2				
58,6							
Et_2O, -78°C, distillation, specific apparatus for manipulating BuLi							

Chem Eur J 2017, 23, 9761-9765 - II STEP				sE-Factor	RME	yield	AE
**Raw materials and reactants (g)**	**mmol**	**MM**	**eq**	8,46	0,106	44,0	0,34
alcohol **4**							
25,9	171,0	152,0					
methanesulphonyl chloride							
21,5	188,1	114,5	1,1				
triethylamine							
43,3	427,5	101,2	2,5				
sodium hydrogencarbonate							
14,4	171,0	84,0					
water							
3,1	171,0	18,0					
sum of raw materials, reactants, catalysts							
108,1	0,0						
**Products [g]**							
purity (%)							
perilly alcohol							
	**yield (%)**						
11,4	**44,0**	152,0	75,2				
sum of total waste							
96,7							
DCM as a solvent, 0°C, 1h, then room temperature, one pot, flash chromatography							

Chem Eur J 2017, 23, 9761-9765	
**2 steps, overall yields =**	**38,3**
**sE-Factor=**	**13,6**
**AE=**	**0,23**
**RME=**	**0,0725**

Our procedure - I step				sE-Factor	RME	yield	AE
**Raw materials and reactants (g)**	**mmol**	**MM**	**eq**	1,55	0,392	41,8	1,00
limonene oxides (cis+trans)							
16,00	105,3	152,0					
Al(*i* -PrO)$_3$							
1,07	5,3	204,2	5,00E-02				
sum of raw materials, reactants, catalysts							
17,07							
**Products [g]**							
crude mixture							
15,20							
molar % compound cis-**4** (NMR)							
**44,0**							
weigth % compound cis-**4** (NMR)							
**44,0**							
compound **4**							
6,69	44,0	152,0					
	**yield (only compound cis-4)**						
	41,8						
sum of total waste							
10,39							
toluene, reflux 5h							

Our procedure - II + III step (column chromatography)				sE-Factor	RME	yield	AE
**Raw materials and reactants (g)**	**mmol**	**MM**	**eq**	11,7	0,0853	33,5	0,34
alcohol **4** (mix of isomers) 44% purity							
15,20	100,0	152,0					
methanesulphonyl chloride							
14,89	130,0	114,5	1,3				
triethylamine							
20,2	200,0	101,2	2,0				
sodium hydrogencarbonate							
12,60	150,0	84,0	1,5				
water							
1,80	100,0	18,0					
sum of raw materials, reactants, catalysts							
64,7							
**Products [g]**							
perillyl alcohol after column chromatography							
5,09	33,5	152,00					
	**yield (only perillyl alcohol)**						
	33,5						
sum of total waste							
59,6							

Our procedure	
**2 steps, overall yields =**	**31,8**
**sE-Factor=**	**13,8**
**AE=**	**0,34**
**RME=**	**0,0764**

Our procedure - I step				sE-Factor	RME	yield	AE
**Raw materials and reactants (g)**	**mmol**	**MM**	**eq**	1,55	0,392	41,8	1,00
limonene oxides (cis+trans)							
16,00	105,3	152,0					
Al(i-PrO)$_3$							
1,07	5,3	204,2	5,00E-02				
sum of raw materials, reactants, catalysts							
17,07							
**Products [g]**							
crude mixture							
15,20							
molar % compound cis-**4** (NMR)							
**44,0**							
weigth % compound cis-**4** (NMR)							
**44,0**							
compound **4**							
6,69	44,0	152,0					
	**yield (only compound cis-4)**						
	41,8						
sum of total waste							
10,39							
toluene, reflux 5h							

Our procedure - II + III step (with column chromatography)				sE-Factor	RME	yield	AE
**Raw materials and reactants (g)**	**mmol**	**MM**	**eq**	11,7	0,0853	33,5	0,34
alcohol **4** (mix of isomers) 44% purity							
15,20	100,0	152,0					
methanesulphonyl chloride							
14,89	130,0	114,5	1,3				
triethylamine							
20,2	200,0	101,2	2,0				
sodium hydrogencarbonate							
12,60	150,0	84,0	1,5				
water							
1,80	100,0	18,0					
sum of raw materials, reactants, catalysts							
64,7							
**Products [g]**							
perillyl alcohol after column chromatography							
5,09	33,5	152,00					
	**yield (only perillyl alcohol)**						
	33,5						
sum of total waste							
59,6							
One step, acetone then water, distillation of final product							

Our procedure (column chromatography)					sE-Factor	RME	yield	AE
**Raw materials and reactants (g)**	**mL**	**mmol**	**MM**	**eq**	4,55	0,180	70,1	0,71
perillyl alcohol)								
**5,1**		33,5	152,0					
acetone								
7,8		133,9	58,0	4,000				
alcohol dehydrogenase (g of protein in ADH lysate)								
3,6	0,0							
buffer	407,2							
Na2HPO4								
2,70								
NaH2PO4								
0,16								
NAD+								
0,19		0,28	663,4					
sum of raw materials, reactants, catalysts								
19,5								
**Products [g]**								
crude mixture								
4,85								
Isolation by distillation								
perillaldehyde								
3,52		23,5	150,0					
	**yield (only perillaldehyde)**							
	70,1							
sum of total waste								
16,0								
One step, acetone then water. Since acetone is both a reagent and a solvent, only the quantity necessary as a								

Our procedure	
**overall yields =**	**22,3**
**sE-Factor=**	**24,4**
**AE=**	**0,29**
**RME=**	**0,0434**

Biorg. Med. Chem 1999					sE-Factor	RME	yield	AE
**Raw materials and reactants (g)**	**mL**	**mmol**	**MM**	**eq**	14,2	0,06593	64,0	0,63
perillyl alcohol)								
**0,1520**		1,000	152,0					
MnO2								
1,3041		15,000	86,9	15,000				
sum of raw materials, reactants, catalysts								
1,456								
**Products [g]**								
perillaldehyde								
0,096		0,640	150,0					
	**yield (only perillaldehyde)**							
	64,0							
sum of total waste								
1,360								
**Oxidation in hexane 22 mL, 2h r.t., then column chromatography**								

Org. Lett 2002					sE-Factor	RME	yield	AE
**Raw materials and reactants (g)**	**mL**	**mmol**	**MM**	**eq**	0,1	0,87349	90,0	0,82
perillyl alcohol)								
**0,152**		1,000	152,0					
TEMPO								
0,0015625		0,010	156,3	0,01				
CuCl								
0,00099		0,010	99,0	0,010				
oxygen (xs)								
			32,0					
ionic liquid	1,50							
sum of raw materials, reactants, catalysts								
0,155								
**Products [g]**								
perillaldehyde								
0,135		0,900	150,0					
	**yield (only perillaldehyde)**							
	90,0							
sum of total waste								
0,020								
The solvent is an ionic liquid. 65° C, 16 h, then column chromatography.								

Org. Lett 2013					sE-Factor	RME	yield	AE
**Raw materials and reactants (g)**	**mL**	**mmol**	**MM**	**eq**	0,1	0,877451	90,0	0,82
perillyl alcohol)								
**0,152**		1,000	152,0					
TEMPO								
0,0015625		0,010	156,3	0,01				
NaCl								
0,0002922		0,005	58,4	0,005				
$Fe(NO_3)_3$ ·9 H2O								
0,0040399		0,010	404,0	0,010				
oxygen (xs)			32,0					
1,2-dichloroetano	10,00							
sum of raw materials, reactants, catalysts								
0,154								
**Products [g]**								
perillaldehyde								
0,135		0,900	150,0					
	**yield (only perillaldehyde)**							
	90,0							
sum of total waste								
0,019								
The solvent is an ionic liquid. 65° C, 16 h, then column chromatography.								

The penalty points to calculate the EcoScale

		Epoxide rearrangement				Allylic displacement				Final oxidation	
**Parameter**	**Penalty points**	**Synth. Commun 1988**	**Tele 2014**	**Chem Eur J 2017**	**this work**	**Synth. Commun 1988**	**Tele 2014**	**Chem Eur J 2017**	**this work**	**Synth. Commun 1988**	**this work**
reaction yield		56,9	60,5	56,6	41,8	61,0	41,7	44,0	31,8	64,0	70,1
**1. Yield**	(100 – %yield)/2	21,6	19,8	21,7	29,1		29,1	28,0	34,1	18,0	15,0
**2. Price of reaction components (to obtain 10 mmol of end product)**											
Inexpensive (< $10)	0			0	0	0	0	0	0		0
Expensive (> $10 and < $50)	3		3							3	
Very expensive (> $50)	5	5									
**3. Safety**											
N (dangerous for environment)	5	40	40	30	20	25	55	30	25	50,00	10
T (toxic)	5										
F (highly flammable)	5										
E (explosive)	10										
F+ (extremely flammable)	10										
T+ (extremely toxic)	10										
**4. Technical setup**											
Common setup	0				0			0	0	0	0
Instruments for controlled addition of chemicals[b]	1	1	1	1		1					
Unconventional activation technique[c]	2										
Pressure equipment, > 1 atm[d]	3						3				
Any additional special glassware	1	1	1	1			1				
(Inert) gas atmosphere	1	1	1	1		1					
Glove box	3										
**5. Temperature/time**											
Room temperature, < 1 h	0										
Room temperature, < 24 h	1										
Heating, < 1 h	2										
Heating, > 1 h	3				3						3
Cooling to 0°C	4	4					4	4	4		
Cooling, < 0°C	5		5	5		5				5	
**6. Workup and purification**											
None	0										
Cooling to room temperature	0				0				0		0
Adding solvent	0		0	0	0		0	0	0	0	0
Simple filtration	0										
Removal of solvent with bp < 150°C	0	0		0	0	0		0	0	0	0
Crystallization and filtration	1										
Removal of solvent with bp > 150°C	2										
Solid phase extraction	2										
Distillation	3										3
Sublimation	3										
Liquid-liquid extraction	3	3	3	3	3	3	3	3	3	3	3
Classical chromatography	10	10	10	10		10	10	10	10	10	
		13,4	16,2	27,3	44,9	55,0	-5,1	25,0	23,9	11,0	66,0

a) Based on the hazard warning symbols. b) Dropping funnel, syringe pump, gas pressure regulator, etc. c) Microwave irradiation, ultrasound or photochemical activation, etc. d) scCO2, high pressure hydrogenation equipment, etc. e) If applicable, the process includes drying of solvent with desiccant and filtration of desiccant.

The penalty points to calculate the EcoScale

		Perillyl alcohol oxidation			
**Parameter**	**Penalty points**	**Biorg. Med. Chem. 1999**	**Org. lett 2002**	**Org. lett 2013**	**this work**
reaction yield		64,0	90	90	70,1
**1. Yield**	(100 – %yield)/2	18,0	5,0	5,0	15,0
**2. Price of reaction components (to obtain 10 mmol of end product)**					
Inexpensive (< $10)	0	0		0	
Expensive (> $10 and < $50)	3				
Very expensive (> $50)	5		5		
**3. Safety**					
N (dangerous for environment)	5	30,00	25	30	10
T (toxic)	5				
F (highly flammable)	5				
E (explosive)	10				
F+ (extremely flammable)	10				
T+ (extremely toxic)	10				
**4. Technical setup**					
Common setup	0	0	0	0	0
Instruments for controlled addition of chemicals[b]	1				
Unconventional activation technique[c]	2				
Pressure equipment, > 1 atm[d]	3				
Any additional special glassware	1				
(Inert) gas atmosphere	1				
Glove box	3				
**5. Temperature/time**					
Room temperature, < 1 h	0				
Room temperature, < 24 h	1	1		1	
Heating, < 1 h	2				
Heating, > 1 h	3		3		3
Cooling to 0°C	4				
Cooling, < 0°C	5				
**6. Workup and purification**					
None	0				
Cooling to room temperature	0		0	0	0
Adding solvent	0				0
Simple filtration	0	0			
Removal of solvent with bp < 150°C	0	0		0	0
Crystallization and filtration	1				
Removal of solvent with bp > 150°C	2				
Solid phase extraction	2				
Distillation	3				3
Sublimation	3				
Liquid-liquid extraction	3	3	3	3	3
Classical chromatography	10	10	10	10	
		38,0	49,0	51,0	66,0

a) Based on the hazard warning symbols. b) Dropping funnel, syringe pump, gas pressure regulator, etc. c) Microwave irradiation, ultrasound or photochemical activation, etc. d) $scCO_2$, high pressure hydrogenation equipment, etc. e) If applicable, the process includes drying of solvent with desiccant and filtration of desiccant.

GC Analyses on a column having a chiral stationary phase for the determination of the enantiomeric excess of (*R*)-perillaldehyde prepared in this work.

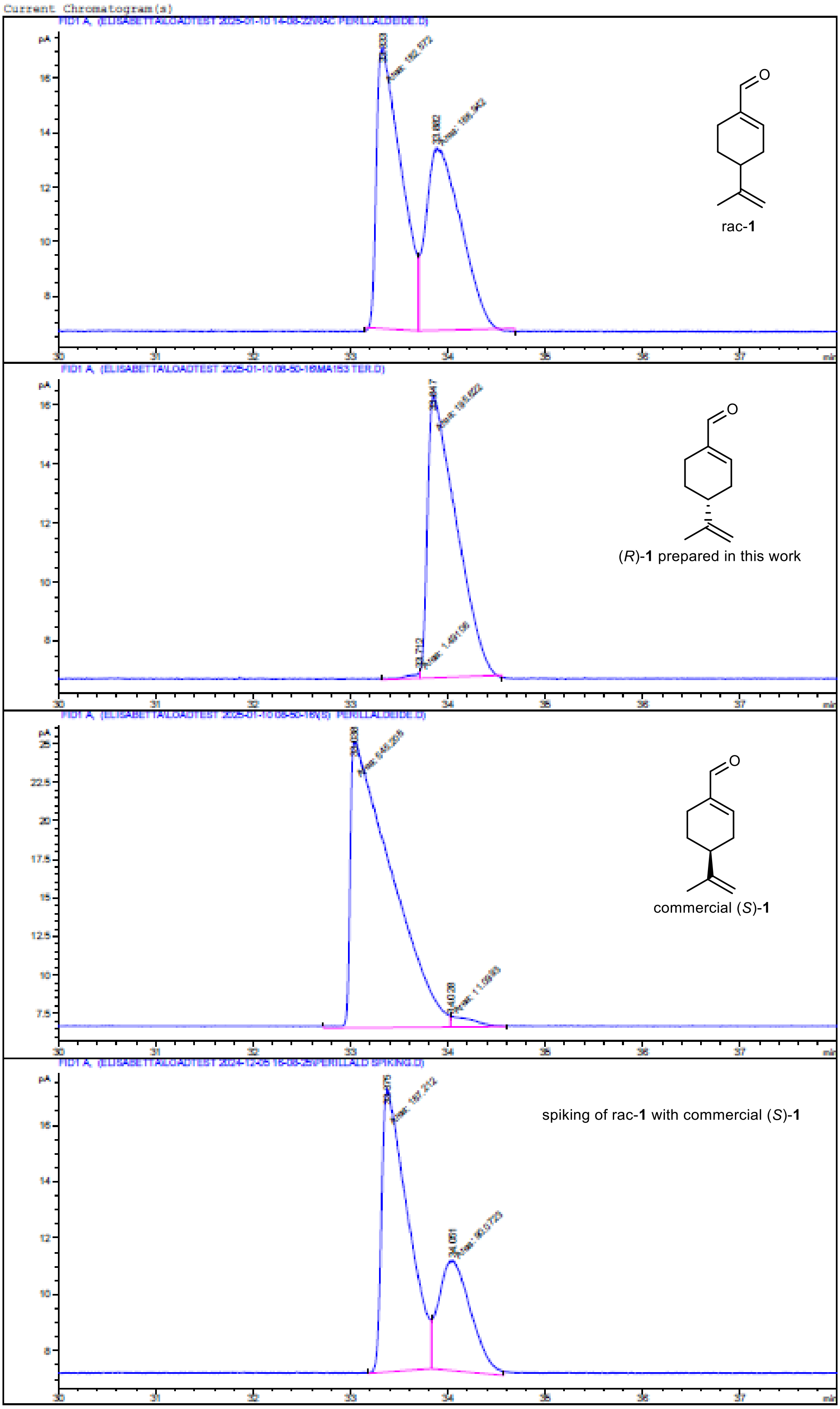

^{1}H and ^{13}C NMR spectra of the mixture obtained by rearrangement of (4*R*)-limonene oxides catalysed by Al(O-*i*-Pr)$_3$

The ^{1}H NMR signals used to calculate the percentage molar composition are shown in the spectrum.

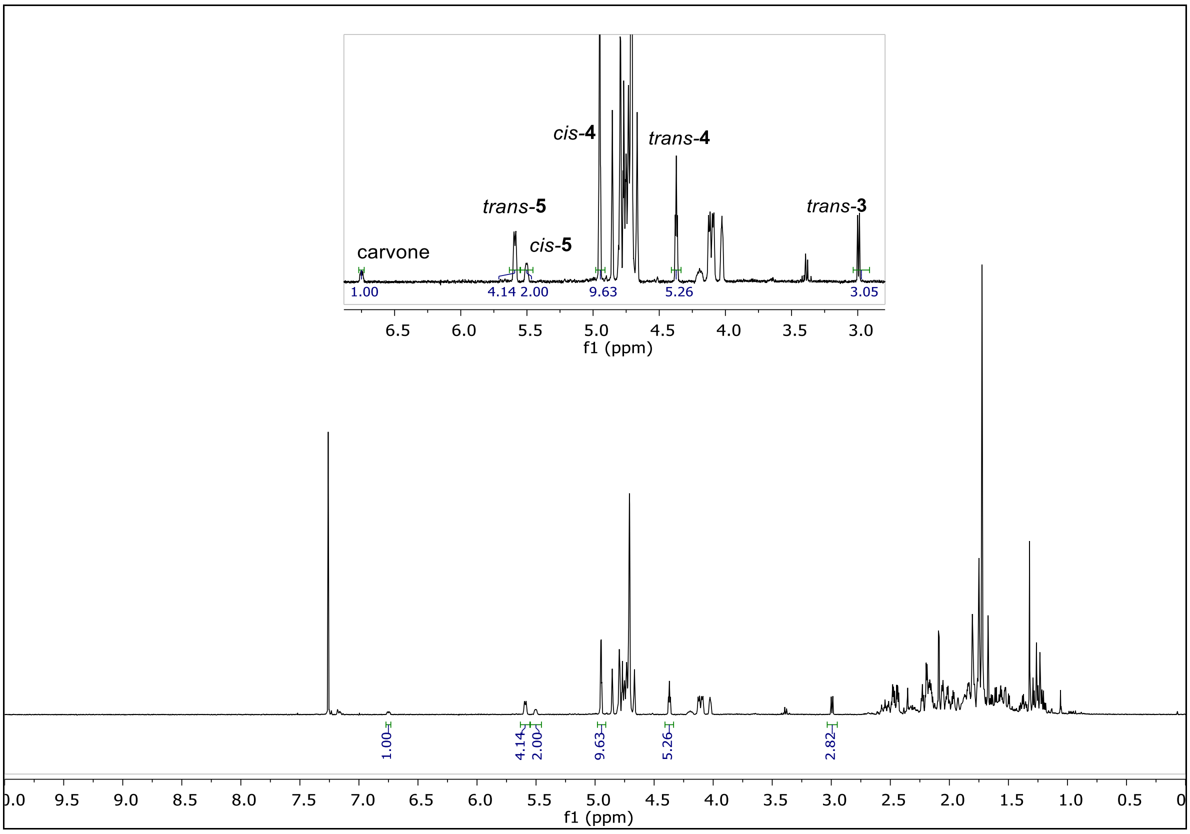

The ^{13}C NMR signals of the **C**HOH of the alcohols *trans*-**4** (72.49 ppm), *cis*-**4** (72.26 ppm), *cis*-**5** (71.02 ppm), and *trans*-**5** (68.66 ppm) and are shown in the spectrum.

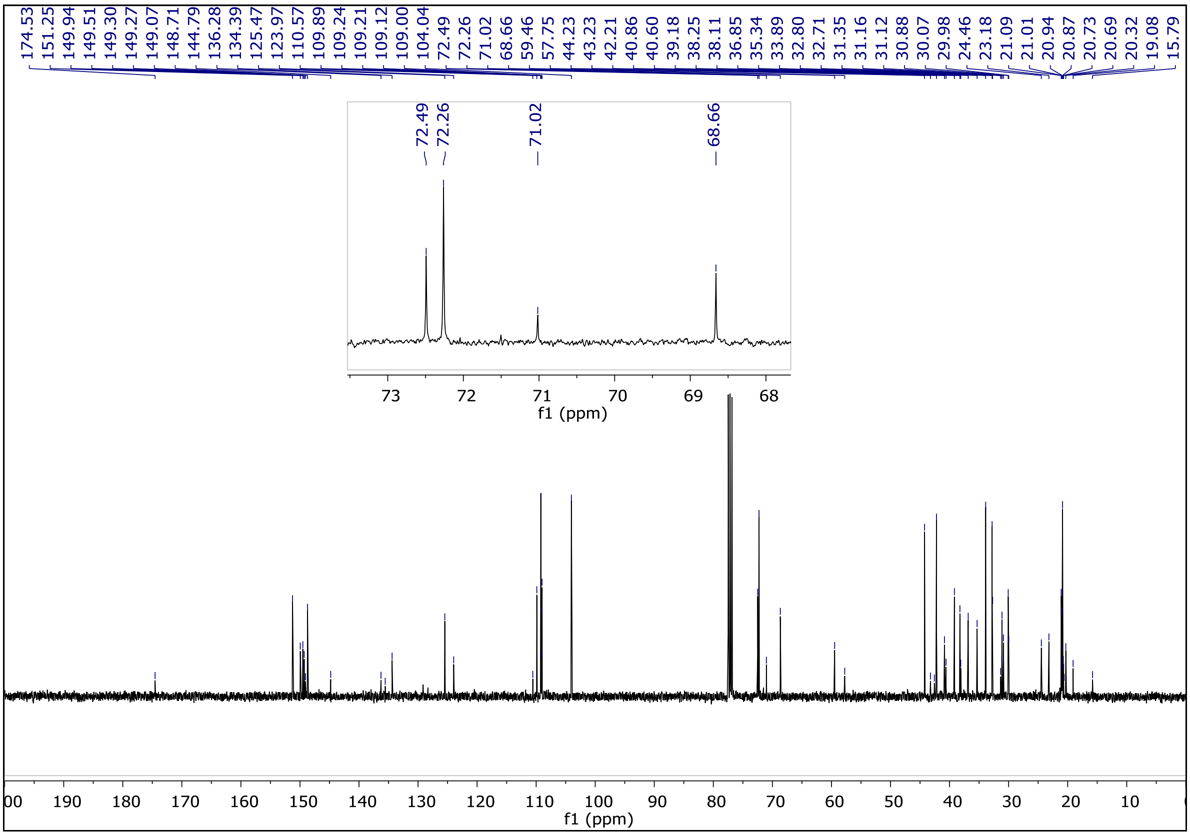

^{1}H and ^{13}C NMR spectra of (*R*)-perillyl alcohol ((*R*)-2)

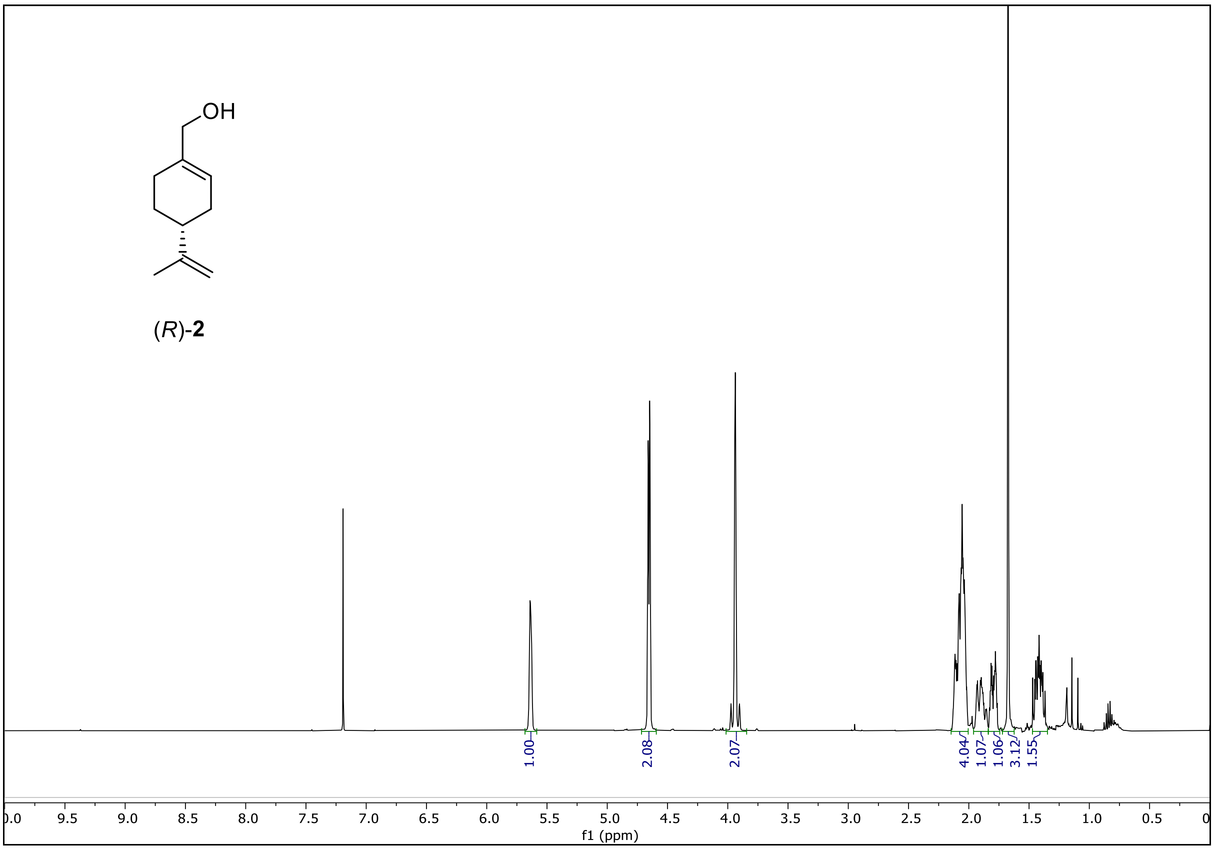

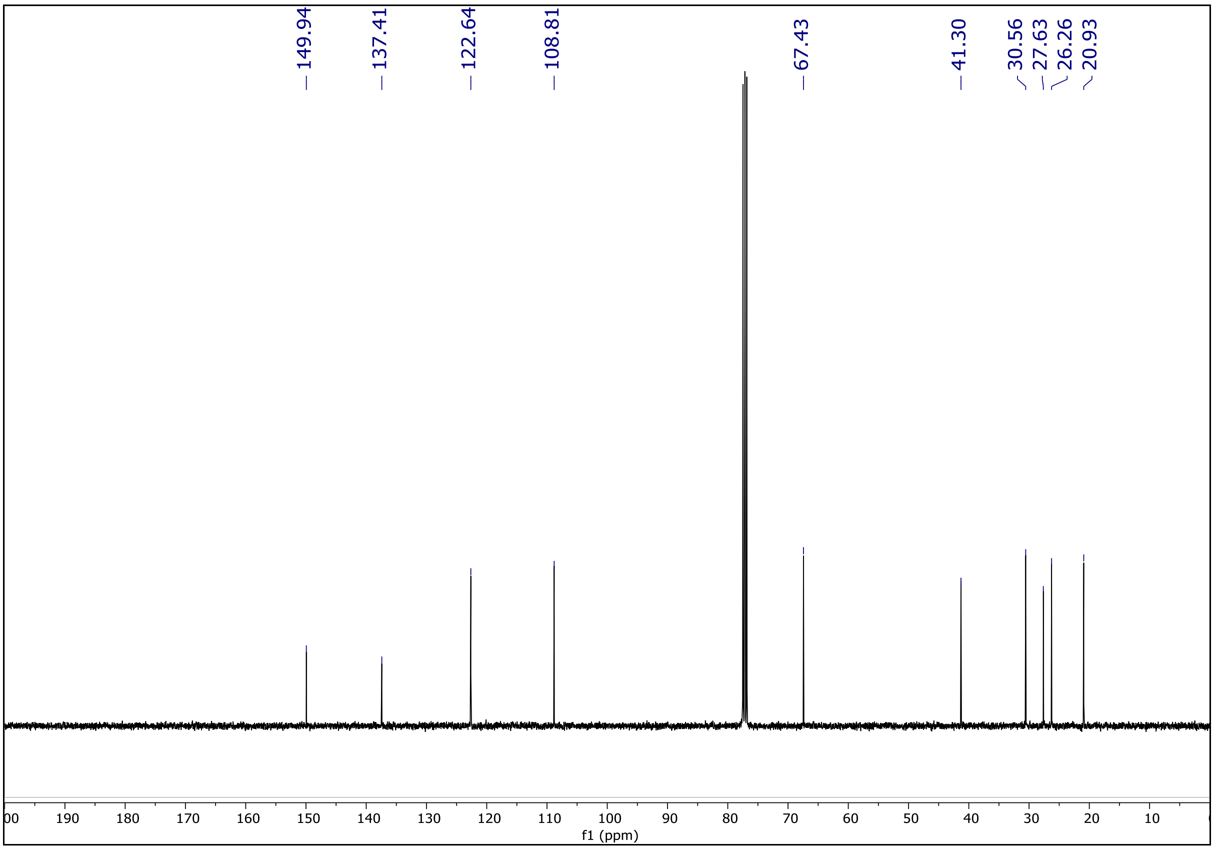

^{1}H and ^{13}C NMR spectra of the mixture obtained by allylic rearrangement by *S$_N$2'* displacement of the mesylate derivatives of alcohols 4 and 5

The ^{1}H NMR signals used to calculate the percentage molar composition are shown in the spectrum.

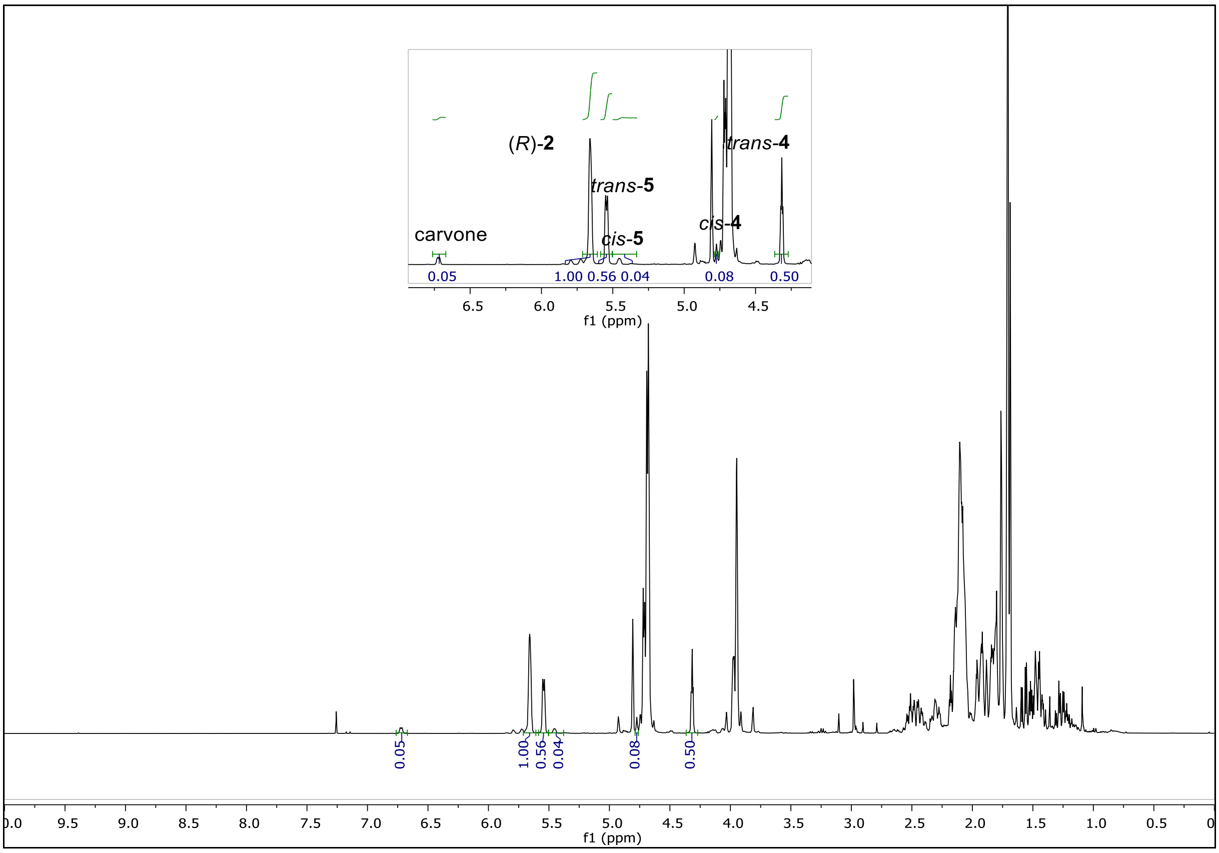

The ^{13}C NMR signals of perillyl alcohol are shown in the spectrum.

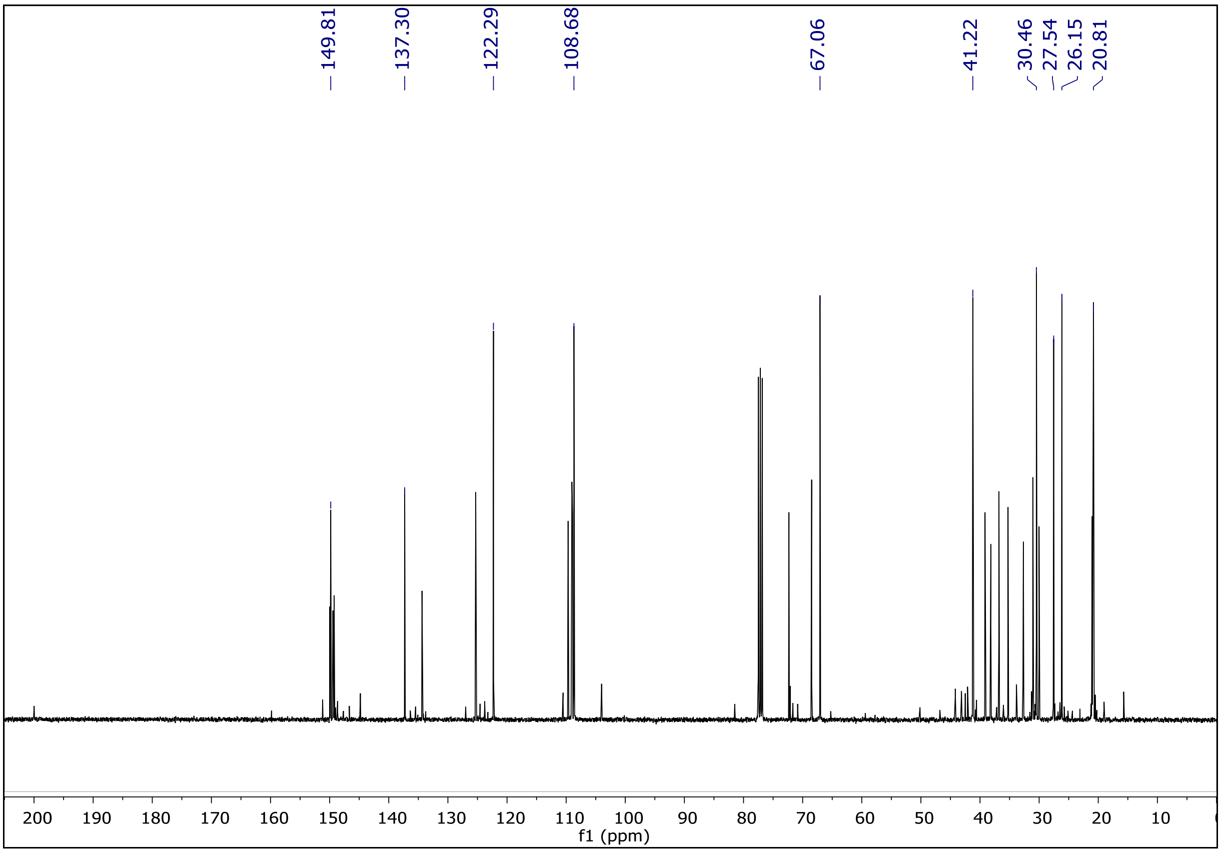

^{1}H and ^{13}C NMR spectra of (*R*)-perillaldehyde ((*R*)-1)

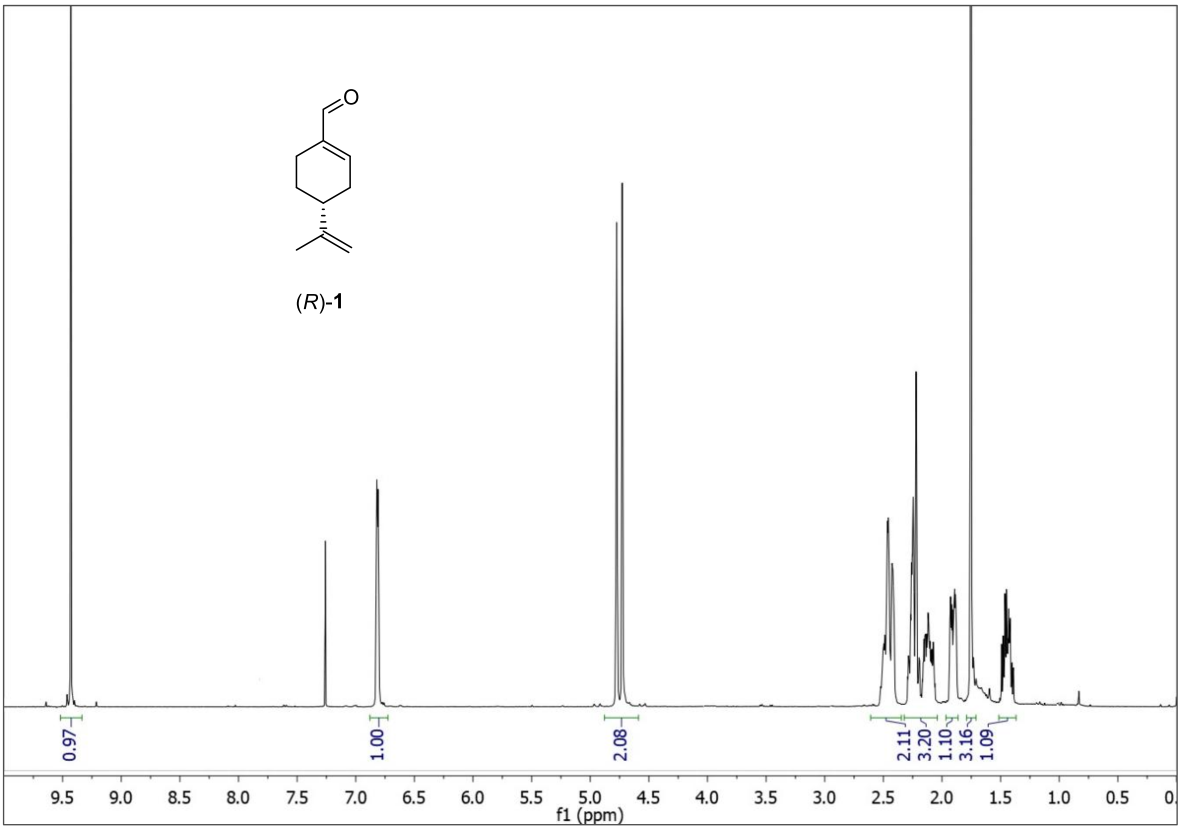

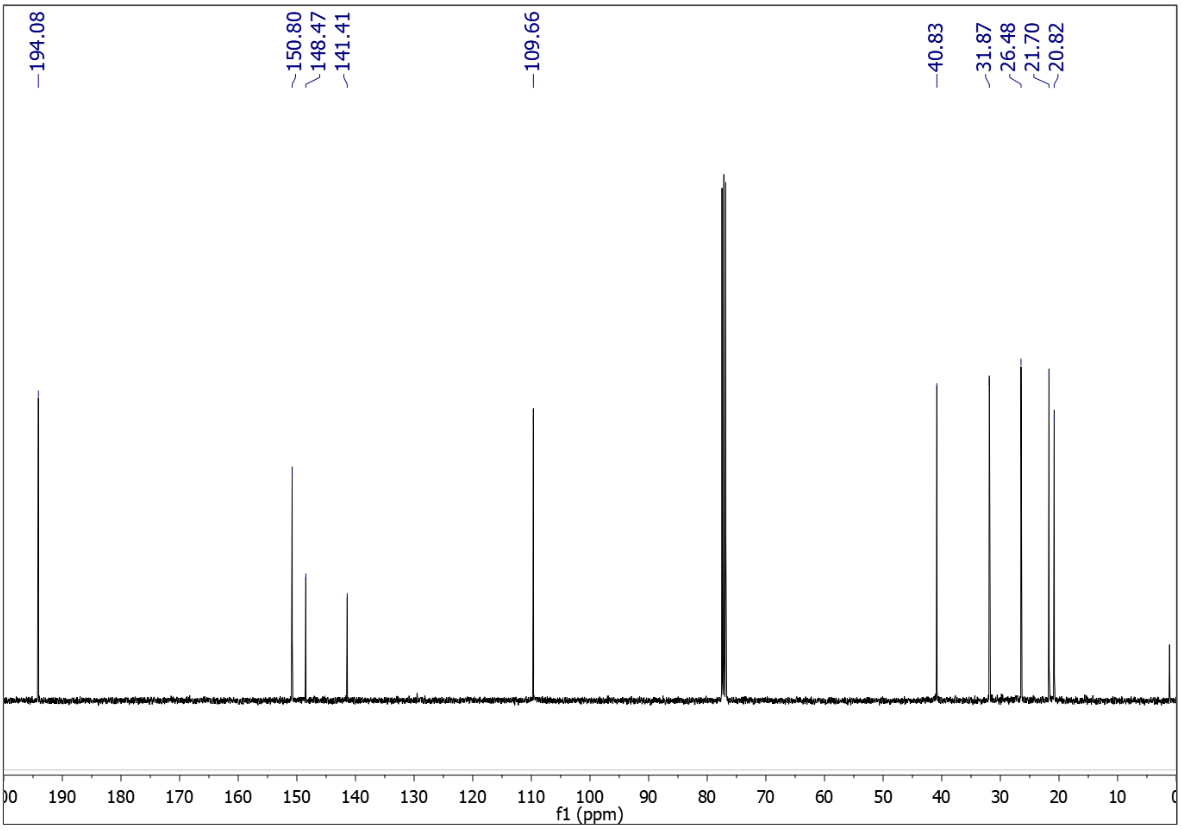

Supplement: Supplementary file 1 [file op5c00340_si_001.pdf]
